# Comparison of Pharmacological Treatment Effects on Long-Time Outcomes in Heart Failure With Preserved Ejection Fraction: A Network Meta-analysis of Randomized Controlled Trials

**DOI:** 10.3389/fphar.2021.707777

**Published:** 2021-11-24

**Authors:** Yaowang Lin, Meishan Wu, Bihong Liao, Xinli Pang, Qiuling Chen, Jie Yuan, Shaohong Dong

**Affiliations:** ^1^ Department of Cardiology, Shenzhen Cardiovascular Minimally Invasive Medical Engineering Technology Research and Development Center, Shenzhen, China; ^2^ Shenzhen People’s Hospital (The Second Clinical Medical College, Jinan University, The First Affiliated Hospital, Southern University of Science and Technology), Shenzhen, China; ^3^ Department of Pharmacy, Shenzhen, China

**Keywords:** heart failure with preserved ejection fraction, all-cause mortality, cardiovascular mortality, HF hospitalization, randomized control trials

## Abstract

Beneficial effects of therapeutic drugs are controversial for heart failure with preserved ejection fraction (HFpEF). This meta-analysis aimed to evaluate and compare the interactive effects of different therapeutic drugs and placebo in patients with HFpEF. A comprehensive search was conducted using PubMed, Google Scholar, and Cochrane Central Register to identify related articles published before March 2021. The primary outcome was all-cause mortality. Secondary outcomes were cardiovascular mortality, heart failure (HF) hospitalization, and worsening HF events. A total of 14 randomized controlled trials, comprising 19,573 patients (intervention group, *n* = 9,954; control group, *n* = 9,619) were included in this network meta-analysis. All-cause mortality, cardiovascular mortality, and worsening HF events among therapeutic drugs and placebo with follow-up of 0.5–4 years were not found to be significantly correlated. The angiotensin receptor neprilysin inhibitor (ARNI) and angiotensin-converting enzyme inhibitor (ACEI) significantly reduced the HF hospitalizations compared with placebo (hazard ratio [HR] 0.73, 95% confidence interval [CI] 0.60–0.87 and HR 0.64, 95% CI 0.43–0.96, respectively), without heterogeneity among studies. The ARNI was superior to angiotensin receptor blocker (ARB) in reducing HF hospitalizations (HR 0.80, 95% CI 0.71–0.91), and vericiguat 10 mg ranked worse than beta-blockers for reducing all-cause mortality in patients with HFpEF (HR 3.76, 95% CI 1.06–13.32). No therapeutic drugs can significantly reduce mortality, but the ARNI or ACEI is associated with the low risk of HF hospitalizations for patients with HFpEF.

**Systematic Review Registration:** https://www.crd.york.ac.uk/prospero/, identifier CRD42021247034

## Introduction

Heart failure with preserved ejection fraction (HFpEF), also referred to as diastolic heart failure, is a heterogeneous clinical syndrome defined by the presence of signs and symptoms of heart failure (HF) with normal left ventricular ejection fraction (LVEF) (typically considered as ≥50%). The HFpEF constitutes greater than 50% of all HF diagnoses and is associated with considerable morbidity and mortality (Redfield, 2017). The prognosis for patients with HFpEF remains poor, with a 1-year mortality of about 10–30% (Dhingra et al., 2014). While significant advances in reducing HF mortality and improving the cardiac function (Li et al., 2020) have been made in treating heart failure with reduced ejection fraction (HFrEF) (Komajda et al., 2018), pharmacological therapies for HFpEF including angiotensin receptor neprilysin inhibitor (ARNI), angiotensin converting enzyme inhibitors (ACEIs), angiotensin receptor blockers (ARBs), beta-blockers (BBs) and mineralocorticoid receptor antagonists (MRAs), have generally been disappointing, with no convincing evidence of mortality or morbidity reduction. A previous study compared the effects of propranolol against placebo on mortality in elderly patients with HFpEF, and the results showed low mortality, with less nonfatal myocardial infarction and higher LVEF in the propranolol group (Aronow et al., 1997). However, the PARAGON HF trial results showed that the use of ARNI in HFpEF did not result in a significantly lower rate of HF hospitalization and cardiovascular mortality. However, subgroup analyses suggested heterogeneity with possible benefit for women patients and patients with LVEF ranged 45–57% (Solomon et al., 2019). The PEP-CHF trial of perindopril and TOPCAT trial of spironolactone in patients with HFpEF showed a significantly lower rate of HF hospitalization in the perindopril and spironolactone groups (Cleland et al., 2006; Pitt et al., 2014).

Owing to inconsistent or lack of adequate evidence concerning beneficial effects of therapeutic drugs in patients with HFpEF, in this network meta-analysis, we aimed to comprehensively review randomized controlled trials (RCTs) of pharmacological treatment in patients with HFpEF to conduct comparison of all medications against placebo and between pharmacological agents on mortality, HF hospitalization, and worsening HF events despite the paucity of head-to-head comparison of therapies in RCTs.

## Materials and Methods

This network-meta-analysis is registered in PROSPERO (CRD42021247034).

### Search Strategy

A comprehensive search was conducted using PubMed, Google Scholar, and Cochrane Central Register to identify related articles published before March 30, 2021 (Supplementary Tables S1A,B). The keywords included *“heart failure with preserved ejection fraction,”* “*diastolic heart failure,*” “*angiotensin receptor neprilysin inhibitor or sacubitril–valsarta,*” “*angiotensin converting enzyme inhibitors,*” “*angiotensin receptor blockers,*” “*beta blockers,*” “*mineralocorticoid receptor antagonists,*” “*digoxin,*” “*phosphodiesterase-5 inhibition or sidenafi,*” “*vericiguat,*” *“sodium-glucose cotransporter-2,”* and “*diuretic.*” Certain additional related publications, such as review articles and editorials, were also assessed.

### Eligibility Criteria

The inclusion criteria were as follows: 1) patients diagnosed with HF; 2) LVEF ≥40%; 3) the treatment and control groups received oral drugs and placebo, respectively, 4) reported outcomes including all-cause mortality, cardiovascular mortality, HF hospitalizations or worsening HF events, 5) RCTs, and 6) articles published in English.

### Study Outcomes

The primary outcome was all-cause mortality. Secondary outcomes included cardiovascular mortality, HF hospitalizations, and worsening HF events (defined by either a decrease by ≥ 1 metabolic equivalent (METs) or an increase by one or more class in New York Heart Association (NYHA) functional class between baseline and months).

### Data Extraction and Quality Assessment

First, two authors (YL and JY) screened the title and abstract independently to identify relevant papers. Inclusion of a study was decided by consensus between the two investigators. Second, standardized pretest tables (including country, population, sample size, age, NYHA, other baseline characteristics, interventions, endpoints and follow-up data) were used to extract data from included studies on full text. The data extraction was independently assessed in a blinded fashion. Any disagreement between the two authors was resolved by discussion. If there was no consensus, a third author (SHD) was consulted.

The Cochrane Collaboration tool was used to assess the risk of bias and the confidence in network meta-analysis (CINeMA) framework was used to assess the indirectness, imprecision, heterogeneity and incoherence of the included RCTs. Funnel plots were drawn to assess the publication bias.

### Statistical Analysis

Statistical analyses were performed using the STATA software, version 14.0 (StataCorp, United States). The combined hazard ratios (HRs) and their corresponding 95% confidence intervals (CIs) were calculated for primary and secondary outcomes. The surface under the cumulative ranking (SUCRA) curve with a rankogram plot was performed to provide a hierarchy of different treatments. Heterogeneity between studies was assessed using the *I* ^*2*^ statistic. If substantial heterogeneity was detected (*I* ^*2*^ > 50%), subgroup analyses were performed to reduce the heterogeneity. Publication bias was evaluated using Begg’s funnel plots. A *p* value <0.05 was considered statistically significant.

## Results

### Characteristics of Included Studies

**FIGURE 1:**
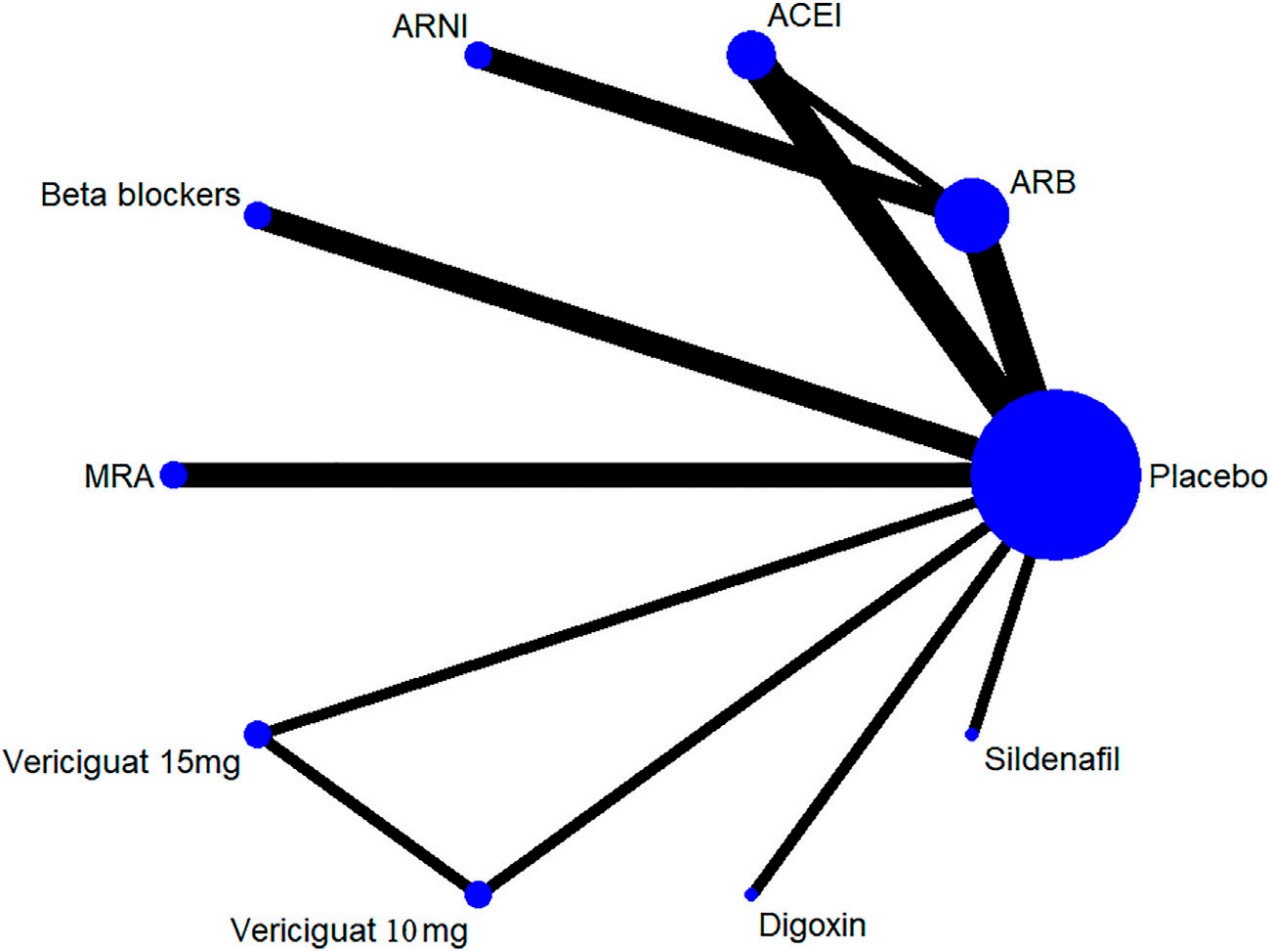
Flow diagram of the study selection process. HF, heart failure; MRA, mineralocorticoid receptor antagonist; SLGT2, sodium-glucose cotransporter-2.

The literature research identified a total of 782 related articles, of which 103 articles were duplicates, and 657 articles did not fulfill the inclusion criteria and were thus excluded. Further, eight articles were excluded as no relevant endpoint data reported. Eventually, 14 RCTs (Aronow et al., 1997; Yusuf et al., 2003; Zi et al., 2003; Ahmed et al., 2006; Cleland et al., 2006; Massie et al., 2008; Yip et al., 2008; Solomon et al., 2012; Edelmann et al., 2013; Redfield et al., 2013; Yamamoto et al., 2013; Pitt et al., 2014; Solomon et al., 2019; Armstrong et al., 2020) (Figure 1) were included in this meta-analysis. In total, 19,573 patients were randomized to either drug intervention group (*n* = 9,954) or control group (*n* = 9,619) with follow-up time duration from 0.5 to 4 years. The mean age in the included studies ranged from 56 to 89. All of the studies were parallel, randomized, and controlled, among which 6 studies were double-blinded, two studies were single-blinded, six studies were open-label studies. Between intervention group and control group, no significant difference was found for the men age and NYHA class III-IV (Table 1). The other baseline characteristics of the included patients were balanced between the two treatment groups with respect to cardiovascular risks, baseline treatments and the baseline echocardiographic measures.

**TABLE 1:** Baseline characteristics of included RCTs.

Study	Year/Country	Study	Intervention group	Control group	Design	Age, y	NYHA III-IV, %	All-cause mortality	Cardiovascular mortality	Heart failure hospitalization	Worsening heart failure events	Follow-up
Wilbert, S	1997 United States	Open-label	Propranolol (*n* = 79)	Placebo (*n* = 79)	LVEF ≥40%, >62 years age	81 ± 8 vs. 81 ± 7	47 vs. 49	44/79 vs. 60/797	—	—	—	35 months
Yusuf, S	2003 Canada	Double-blind	Candesartan (*n* = 1,514)	Placebo (*n* = 1,509)	CHARM preserved trial, LVEF ≥40%	67.2 ± 11.1 vs. 67.1 ± 11.1	38.5 vs. 40	—	170/1,514 vs. 170/1,509	241/1514 vs.276/1,509	—	36.6 months
Min Zi	2003 United Kingdom	Double-blind	Quinapril (*n* = 36)	Placebo (*n* = 38)	LVEF ≥40%, >62 years age	77 ± 7 vs. 78 ± 7	16.7 vs. 26.3	1/36 vs. 1/38	1/36 vs. 1/38	2/36 vs. 5/38	0/36 vs. 4/38	6 months
Cleland, JG	2006 United Kingdom	Double-blind	Perindopril (*n* = 424)	Placebo (*n* = 426)	PEP-CHF, LVEF ≥40%, >70 years age	75 (72,79) vs. 75 (72,79)	23 vs. 26	17/424 vs. 19/4,267	10/424 vs. 17/426	34/424 vs. 53/426	59/424 vs. 71/426	12 months
Massie,BM	2008 United States	Single-blind	Irbesartan (*n* = 2067)	Placebo (*n* = 2061)	I- PRESERVE, LVEF ≥45%, >60 years age	72 ± 7 vs. 72 ± 7	80 vs. 79	445/2067 vs. 436/2061	311/2067 vs. 302/2061	325/2067 vs. 336/2061	291/2067 vs. 314/2061	49.5 months
Yip, GWK	2008 Hong Kong	Open-label	Irbesartan (*n* = 53) vs Ramipril (*n* = 39)	Placebo (*n* = 47)	HK-PROBE, LVEF ≥45%, >18 years age	75 ± 8.5 vs. 74 ± 6.1 vs. 73 ± 8.4	30.4 vs. 33.3 vs. 28.0	1/53 vs. 0/39 vs. 3/47	1/53 vs. 0/39 vs. 1/47	6/53 vs. 5/39 vs. 6/47	—	12 months
Solomon,SD	2012 United States	Double-blind	Sacubitril–valsartan (*n* = 149)	Valsartan (*n* = 152)	PARAMOUNT, LVEF ≥45%, >18 years age	70.9 ± 9.4 vs. 71.2 ± 8.9	19 vs. 21	1/149 vs. 2/152	1/149 vs. 2/152	—	9/149 vs. 12/152	36 weeks
Edelmann, F	2013 Austria	Double-blind	Spironolactone (*n* = 213)	Placebo (*n* = 209)	Aldo-DHF, LVEF ≥50%, >18 years age	67 ± 8 vs. 67 ± 8	15 vs. 12	1/213 vs. 0/209	1/213 vs. 0/2091	21/213 vs. 15/209	—	12 months
Yamamoto,K	2013 Japan	Open-label	Carvedilol (*n* = 120)	Placebo (*n* = 125)	J- DHF, LVEF >40%, >20 years age	73 ± 10 vs. 71 ± 11	15 vs. 8	18/120 vs. 21/1,257	8/120 vs. 7/1,255	21/120 vs. 27/125	25/120 vs. 31/125	24 months
Pitt, B	2014 United States	Double-blind	Spironolactone (*n* = 1722)	Placebo (*n* = 1723)	TOPCAT, LVEF ≥45%, >18 years age	68.7 (61,76.4) vs. 68.7 (60.7,75.5)	33.4 vs. 32.6	252/1722 vs. 274/1723	160/1722 vs. 176/1723	206/1722 vs. 245/1723	—	3.3 years
Solomon, SD	2019 United Kingdom	Single-blind	Sacubitril–valsartan (*n* = 2,407)	Valsartan (*n* = 2,389)	PARAGON-HF, LVEF ≥45%, >18 years age	72.7 ± 8.3 vs. 72.5 ± 8.5	19.3 vs. 20.3	342/2,407 vs. 349/2,389	204/2,407 vs. 212/2,389	690/2,407 vs. 797/2,389	202/2,407 vs. 221/2,389	4 years
Armstrong, PW	2020 Canada	Open-label	Vericiguat 15 mg (*n* = 264) vs. Vericiguat 10 mg (*n* = 262)	Placebo (*n* = 262)	VITALITY-HFpEF, LVEF ≥45%, >45 years age	73.1 ± 9.1 vs. 72.2 ± 9.7 vs. 72.8 ± 9.4	42.4 vs. 41.4 vs. 40.5	10/264 vs. 15/262 vs. 7/262	8/264 vs. 12/262 vs. 4/262	—	—	24 weeks
Ahmed, A	2006 United States	Open-label	Digoxin (*n* = 492)	Placebo (*n* = 496)	LVEF ≥45%, >45 years age	66.7 ± 10.7 vs. 66.9 ± 9.9	21.5 vs. 22.6	115/492 vs. 116/496	81/492 vs. 81/496	61/492 vs. 73/496	89/492 vs. 108/496	37 months
Redfield, MM	2013 United States	Open-label	Sildenafil (*n* = 113)	Placebo (*n* = 103	LVEF ≥50%, >18 years age	68 (62,77) vs. 69 (62,77)	51 vs. 55	3/113 vs. 0/103	2/113 vs. 0/103	15/113 vs. 13/103	—	24 weeks

HF, heart failure; HFpEF, heart failure with preserved ejection fraction; LVEF, left ventricular ejection fraction; “/” = no data available.

### Primary and Secondary Endpoint

**FIGURE 2:**
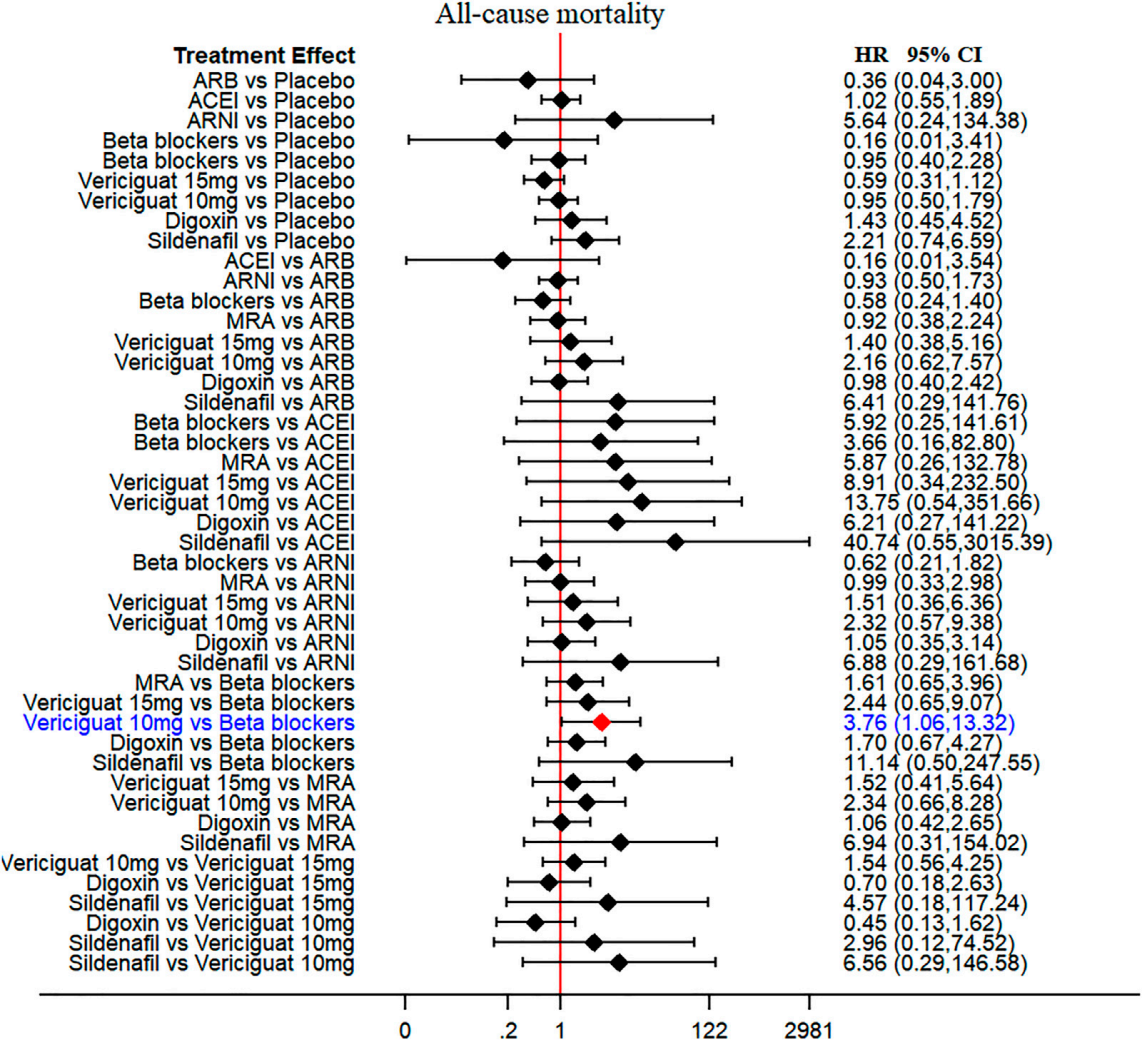
Drug strategies in the Network. The width of lines between each drug reflects the number of studies available for each comparison. ACEI, angiotensin-converting enzyme inhibitor; ARB, angiotensin receptor blocker; ARNI, angiotensin receptor neprilysin inhibitor; MRA, mineralocorticoid receptor antagonist.

**FIGURE 3:**
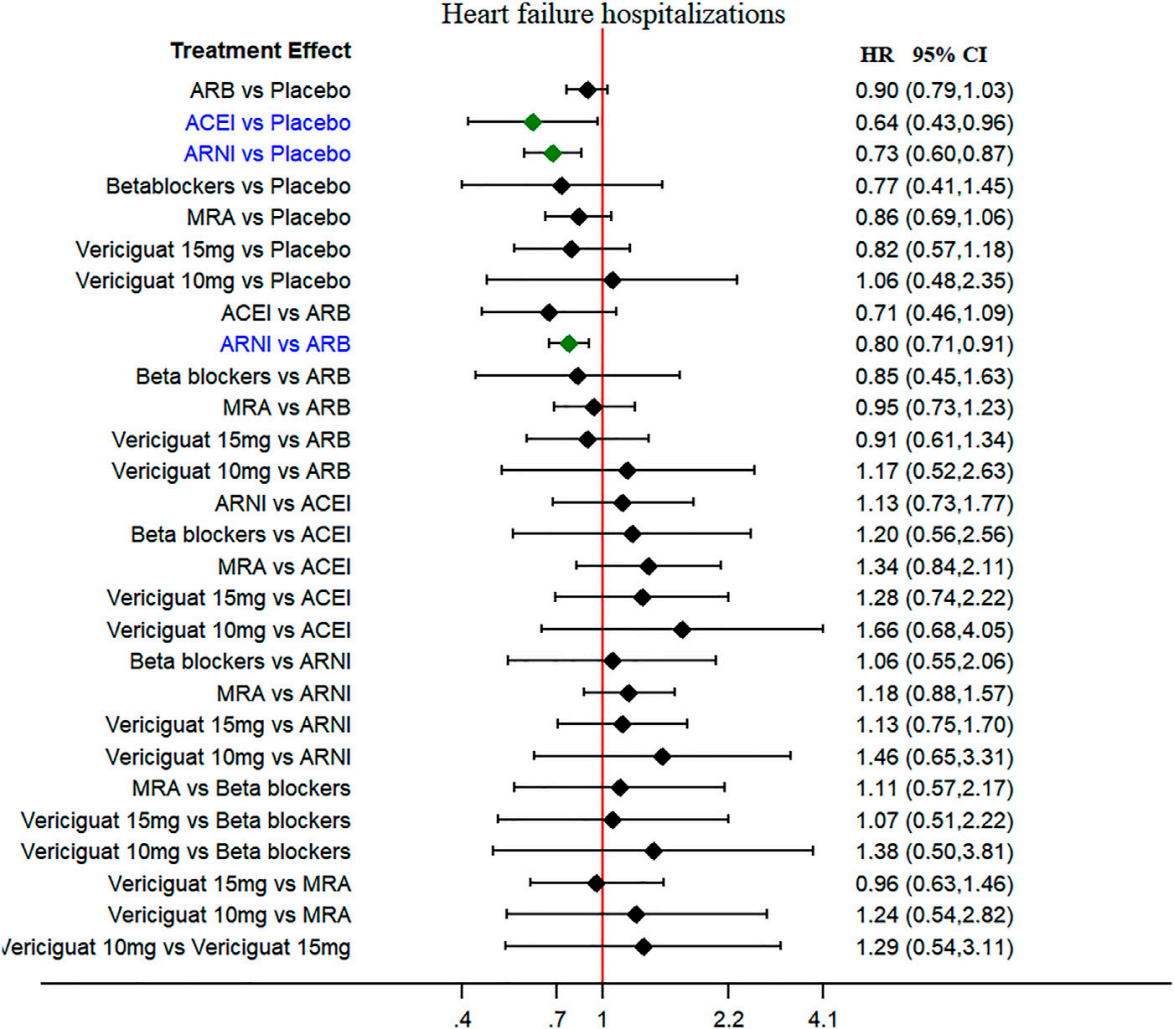
All-cause mortality (primary outcome): Forest plot (estimates as hazard ratio) - All trials.

Thirteen of 14 included RCTs (excluding Yusuf S et al. study) reported all-cause mortality data (primary outcome). Similarly, 13 RCTs (excluding Wilbert S et al. study) reported cardiovascular mortality data (secondary outcome). The drug strategies in the network and all-cause mortality (SUCRA curve with a rankogram plot) are shown in Figure 2 and Supplementary Figure S1), respectively. Network plot of the all-cause mortality among all trials is shown in Supplementary Figure S2. ACEIs (perindopril of PEP-CHF study with LVEF ≥40%, ramipril of HK-PROBE study with LVEF >45%, quinapril of Min Zi study with LVEF ≥40%), ARBs (candesartan of CHARM trial with LVEF ≥40%, irbesartan of I-Preserve study with LVEF >45%, irbesartan of HK-PROBE study with LVEF >45%), ARNI (PARAMOUNT and PARAGON-HF studies with LVEF ≥45%), beta-blockers (carvedilol of J-DHF with LVEF >40%), MRAs (spironolactone of Aldo-DHF with LVEF ≥50% and TOPCAT with LVEF ≥45%), digoxin (digoxin of Ahmed A study with LVEF ≥45%), and sildenafil (sildenafil of Redfield MM study with LVEF ≥50%) were failed to reduce all-cause mortality and cardiovascular mortality among all compared therapeutic drugs and placebo with a follow-up duration of 0.5–4 years [Figure 3 and Supplementary Figure S3]. Propranolol of Wilbert S study with LVEF ≥40% alone showed the positive result of reducing mortality. However, vericiguat 10 mg ranked worse than beta-blocker for reducing all-cause mortality in patients with LVEF ≥45% (HR 3.76, 95% CI 1.06–13.32) (Figure 3).

**FIGURE 4:**
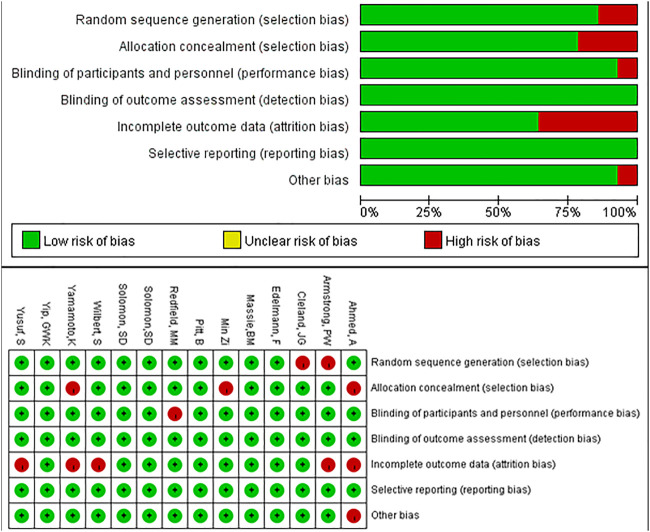
Heart failure hospitalization (secondary outcome): Forest plot (estimates as hazard ratio) -All trials.

The HF hospitalization was reported in 11 studies, and worsening HF events were reported in seven studies (secondary outcomes). The ARNI and ACEI were associated with low risk of HF hospitalizations when compared to placebo (HR 0.73, 95% CI 0.60–0.87 and HR 0.64, 95% CI 0.43–0.96, respectively), without heterogeneity among studies (Figure 4). The ARNI was superior to ARBs in reducing HF hospitalizations risk (HR 0.80, 95% CI 0.71–0.91). No therapeutic drugs did significantly reduce the worsening HF events compared with placebo (Supplementary Figure S4).

### Risk of Bias Assessment and Publication Bias

**FIGURE 5:**
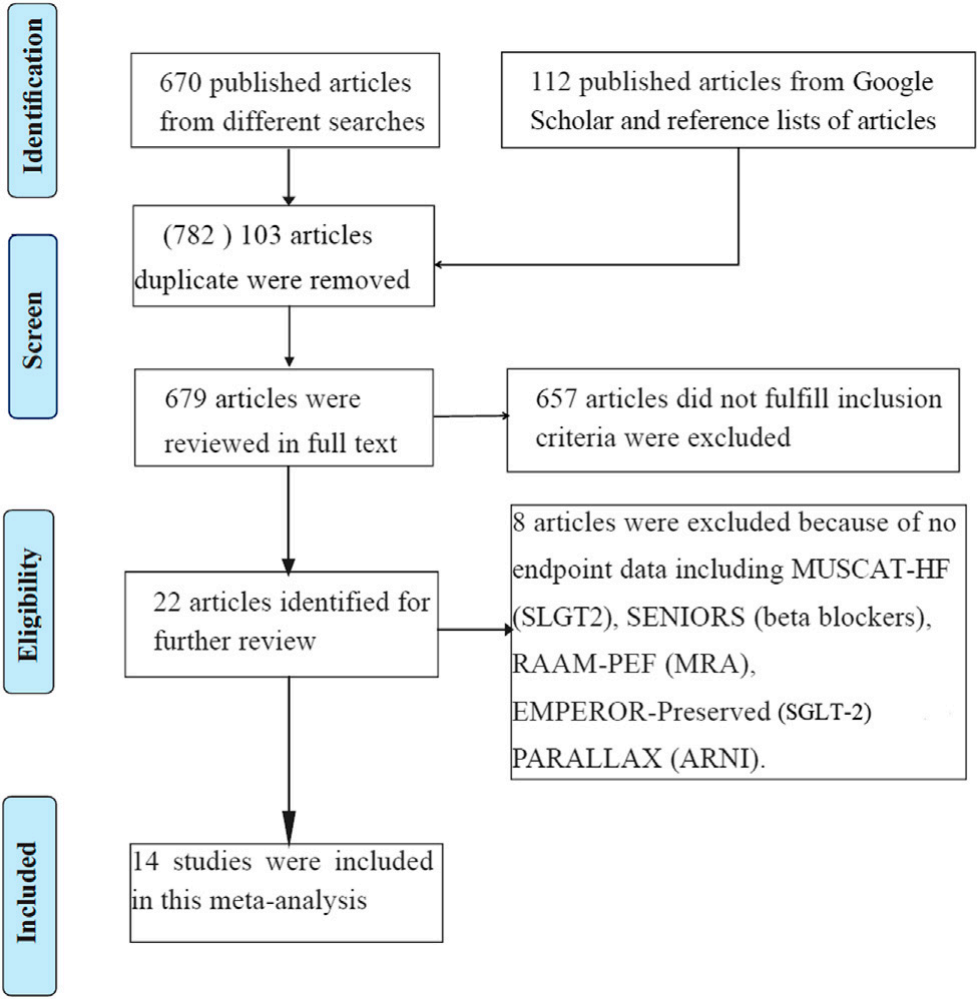
Risk of bias of all trials.

We used the Cochrane Collaboration tool in Review Manager 5.3 to perform quality assessments. Five of the entries had an overall high risk of attrition bias and selection bias, and one study had a high risk of performance bias and other bias, respectively. The remaining studies had low risk of bias (Figure 5). The funnel plot was symmetrical, indicating no evidence of publication bias (Supplementary Figure S5). From the CINeMA framework, the risk of bias contributions of the included studies was showed as Supplementary Figure S6. The imprecision, heterogeneity and incoherence of the mix evidence of included studies were low, while those of the indirect evidence of the included studies were low to moderate (Supplementary Data Sheet S3–5).

## Discussion

In this systematic review and network meta-analysis found that no statistical differences in all-cause and cardiovascular mortality among ACEIs, ARBs, ARNI, MRAs, beta-blockers, vericiguat, digoxin, sildenafil, and placebo, but vericiguat 10 mg ranked worse than beta-blockers for reducing all-cause mortality in patients with HFpEF; 2) The ARNI or ACEI had shown the favorable beneficial effect of significantly reducing HF hospitalization risk when compared to placebo, and ARNI was superior to ARBs in reducing HF hospitalizations.

The pathophysiology of HFpEF is heterogeneous, with multiple individual mechanisms frequently coexisting within the same patient to cause symptomatic HF. The possible pathophysiological mechanisms may include as follow: 1) increased ventricular filling pressure, manifested by thickening of the left ventricular wall and/or left atrial enlargement (Zile et al., 2004; Borlaug and Paulus, 2011), 2) pulmonary vascular disease or dysfunction, and right ventricular failure (Lam et al., 2009), and 3) expansion of plasma volume (Obokata et al., 2017). Although HFpEF is defined as HF patients with LVEF ≥50% (Ponikowski et al., 20162016), LVEF ranged 40–49% was often included in the previous HFpEF RCTs, leading to the methodological limitation of the network meta-analysis.

Identifying effective medical therapies for patients with HFpEF remains a challenge for clinicians. In this network meta-analysis, ACEIs, ARBs, ARNI, MRAs, digoxin, and sildenafil have failed to reduce the risk of mortality in patients with HFpEF or HF with mid-range (borderline) ejection fraction. Only propranolol of Wilbert S study showed a positive result in reducing mortality in patients with LVEF ≥40%. In contrast, vericiguat 10 mg ranked worse than beta-blocker for reducing all-cause mortality in patients with LVEF ≥45%. Nitric oxide is an important regulator of cardiac function, and restoration of nitric oxide-mediated signaling has been proposed as an important treatment target in HF. However, nitric oxide has not been considered the essential factor in the progression of HFpEF compared with HFrEF patients in the VICTORIA trial (Armstrong et al., 2020). Further, renin-angiotensin-aldosterone system (RAAS) inhibitors are commonly foundation of the evidence-based therapies at reducing morbidity and mortality in patients with HFrEF (Komajda et al., 2018). However, there is no tangible evidence for improving the prognosis of HFpEF by RAAS inhibitors (Kuno et al., 2020). From epidemiological studies, older age and higher prevalence of women have been recognized as more frequent in HFpEF compared with HFrEF. Accordingly, the activation of RAAS in HFpEF is lower and therapies based on this mechanism will not reduce the mortality. Instead, tissue congestion caused by high cardiac filling pressures plays a central role in the pathophysiology of HFpEF.

According to the guidelines of the European Society of Cardiology (2016), it is crucial to reduce the readmission burden for patients with HFrEF (Ponikowski et al., 2016). Given that patients with HFpEF tend to be older than their HFrEF counterparts and are limited by disabling symptoms with poor quality of life (Fukuta et al., 2016), rehospitalization burden for HF, being related to a poor quality of life and increased mortality (Gheorghiade et al., 2013), is of great importance between HFrEF and HFpEF. The Medicare represented the 30-day readmission rate for HF was about 23.0% (Mozaffarian et al., 2015). Another study reported that the HF rehospitalization was about 67.4%, of which 35.8% died in-hospital within 1 year (Bueno et al., 2010). In the present network meta-analysis, ARNI or ACEI was found to be significantly decreasing the risk of HF hospitalization compared with placebo. The PEP-CHF study results showed that perindopril significantly reduced the risk of HF rehospitalizations for patients with LVEF ≥40% in the first year, compared with placebo (Cleland et al., 2006). The PARAGON-HF study, comparing ARNI with ARB in patients with LVEF ≥45%, showed no significant reduction of HF hospitalizations (690/2,407 vs. 797/2,389). However, patients taking ARNI was appeared to have an improvement in the NYHA Functional class (HR 1.45, 95% CI 1.13–1.86) (Solomon et al., 2019). The TOPCAT study showed a significant reduction of HF hospitalizations (206/1722 vs. 245/1723) (Dhingra et al., 2014), while the Aldo-DHF study showed no reduction of HF hospitalizations (21/213 vs. 15/209) (Edelmann et al., 2013). The discrepant results could be explained by the different inclusion criteria of study populations with LVEF ≥45% or ≥50%, and different N-terminal prohormone brain natriuretic peptide (NT-proBNP) level at baseline, respectively, in these trials. Frustration over the negative results has led some to suggest that more stringent inclusion criteria, such as high levels NT-proBNP or high NYHA Functional class, are needed for future randomized clinical trials.

The natriuretic peptides (NPs) signaling (down-regulation of NP–cyclic guanosine monophosphate–protein kinase G signaling pathway in the cardiomyocyte) appears to be important in promoting myocardial and vascular stiffness in HFpEF (Greene et al., 2013). Natriuretic peptides also exert anti-hypertrophic and anti-fibrotic effects in the cardiovascular system, and neprilysin inhibition (Sacubitril) in profibrotic signaling has prognostic value in HF (Zile et al., 2019). The ARNIs can significantly reduce NT-proBNP and increase BNP levels. The PARAMOUNT study showed that ARNI reduced NT-proBNP (about 23%) and significantly decreased left atrial volume index after 12 weeks, compared with valsartan. However, no between-group difference was found after 36 weeks in patients with HFpEF (Solomon et al., 2012).

There are several potential limitations in this meta-analysis. First, HFpEF is defined as HF patients with LVEF ≥50%. However, HF patients with LVEF 40–49% (HFmEF) are usually included in the RCTs of HFpEF. It should also be noted that the patients of the included RCTs may not represent the real-life situation by the intrinsic selective bias such as enrolled patients being relatively younger and having fewer comorbidities. The characteristics of design or methodology may also impact or influence the application or interpretation of the results of the study. Second, the studies including MUSCAT-HF (sodium-glucose cotransporter-2), SENIORS (beta-blockers), RAAM-PEF (MRA), and EMPEROR-Preserved (SGLT-2) were not included in this meta-analysis as no relevant endpoint data were reported. Third, the study endpoints such as 6-min walking distance and quality of life measure by the Kansas City Cardiomyopathy Questionnaire were not included in this meta-analysis. Four, lack of application of meta regression and subgroup analyses of heterogeneity disposal in the network meta-analysis. Finally, our meta-analysis does suggest a potential ARNI treatment for reducing HF hospitalization in HFpEF patients. Further randomized trials with stringent inclusion criteria (EF ≥ 50% and high level of BNP) should be conducted to determinate the effects of ARNI on mortality and quality of life in HFpEF. Additionally, more specific types of subpopulations of HFpEF (such as cardiac amyloidosis) and well-established animal models of HFpEF should lead to improvements in outcomes from future trials.

## Conclusion

No therapeutic drugs can significantly reduce mortality, but the ARNI and ACEI have shown a favorable benefit of significantly reducing HF hospitalization risk in patients with HFpEF.

## Data Availability

The raw data supporting the conclusion of this article will be made available by the authors, without undue reservation.

## References

[B1] Ahmed A. Rich M. W. Fleg J. L. Zile M. R. Young J. B. Kitzman D. W. (2006). Effects of Digoxin on Morbidity and Mortality in Diastolic Heart Failure: the Ancillary Digitalis Investigation Group Trial. Circulation 114 (5), 397–403. 10.1161/CIRCULATIONAHA.106.628347 16864724 PMC2628473

[B2] Armstrong P. W. Lam C. S. P. Anstrom K. J. Ezekowitz J. Hernandez A. F. O'Connor C. M. (2020). Effect of Vericiguat vs Placebo on Quality of Life in Patients with Heart Failure and Preserved Ejection Fraction: The VITALITY-HFpEF Randomized Clinical Trial. Jama 324 (15), 1512–1521. 10.1001/jama.2020.15922 33079152 PMC7576403

[B3] Aronow W. S. Ahn C. Kronzon I. (1997). Effect of Propranolol versus No Propranolol on Total Mortality Plus Nonfatal Myocardial Infarction in Older Patients with Prior Myocardial Infarction, Congestive Heart Failure, and Left Ventricular Ejection Fraction > or = 40% Treated with Diuretics Plus Angiotensin-Converting Enzyme Inhibitors. Am. J. Cardiol. 80 (2), 207–209. 10.1016/s0002-9149(97)00320-2 9230162

[B4] Borlaug B. A. Paulus W. J. (2011). Heart Failure with Preserved Ejection Fraction: Pathophysiology, Diagnosis, and Treatment. Eur. Heart J. 32 (6), 670–679. 10.1093/eurheartj/ehq426 21138935 PMC3056204

[B5] Bueno H. Ross J. S. Wang Y. Chen J. Vidán M. T. Normand S. L. (2010). Trends in Length of Stay and Short-Term Outcomes Among Medicare Patients Hospitalized for Heart Failure, 1993-2006. Jama 303 (21), 2141–2147. 10.1001/jama.2010.748 20516414 PMC3020983

[B6] Cleland J. G. Tendera M. Adamus J. Freemantle N. Polonski L. Taylor J. (2006). The Perindopril in Elderly People with Chronic Heart Failure (PEP-CHF) Study. Eur. Heart J. 27 (19), 2338–2345. 10.1093/eurheartj/ehl250 16963472

[B7] Dhingra A. Garg A. Kaur S. Chopra S. Batra J. S. Pandey A. (2014). Epidemiology of Heart Failure with Preserved Ejection Fraction. Curr. Heart Fail. Rep. 11 (4), 354–365. 10.1007/s11897-014-0223-7 25224319

[B8] Edelmann F. Wachter R. Schmidt A. G. Kraigher-Krainer E. Colantonio C. Kamke W. (2013). Effect of Spironolactone on Diastolic Function and Exercise Capacity in Patients with Heart Failure with Preserved Ejection Fraction: the Aldo-DHF Randomized Controlled Trial. Jama 309 (8), 781–791. 10.1001/jama.2013.905 23443441

[B9] Fukuta H. Goto T. Wakami K. Ohte N. (2016). Effects of Drug and Exercise Intervention on Functional Capacity and Quality of Life in Heart Failure with Preserved Ejection Fraction: A Meta-Analysis of Randomized Controlled Trials. Eur. J. Prev. Cardiol. 23 (1), 78–85. 10.1177/2047487314564729 25520380

[B10] Gheorghiade M. Vaduganathan M. Fonarow G. C. Bonow R. O. (2013). Rehospitalization for Heart Failure: Problems and Perspectives. J. Am. Coll. Cardiol. 61 (4), 391–403. 10.1016/j.jacc.2012.09.038 23219302

[B11] Greene S. J. Gheorghiade M. Borlaug B. A. Pieske B. Vaduganathan M. Burnett J. C. Jr. (2013). The cGMP Signaling Pathway as a Therapeutic Target in Heart Failure with Preserved Ejection Fraction. J. Am. Heart Assoc. 2 (6), e000536. 10.1161/JAHA.113.000536 24334823 PMC3886746

[B12] Komajda M. Böhm M. Borer J. S. Ford I. Tavazzi L. Pannaux M. (2018). Incremental Benefit of Drug Therapies for Chronic Heart Failure with Reduced Ejection Fraction: a Network Meta-Analysis. Eur. J. Heart Fail. 20 (9), 1315–1322. 10.1002/ejhf.1234 29806165

[B13] Kuno T. Ueyama H. Fujisaki T. Briasouli A. Takagi H. Briasoulis A. (2020). Meta-Analysis Evaluating the Effects of Renin-Angiotensin-Aldosterone System Blockade on Outcomes of Heart Failure with Preserved Ejection Fraction. Am. J. Cardiol. 125 (8), 1187–1193. 10.1016/j.amjcard.2020.01.009 32081366

[B14] Lam C. S. Roger V. L. Rodeheffer R. J. Borlaug B. A. Enders F. T. Redfield M. M. (2009). Pulmonary Hypertension in Heart Failure with Preserved Ejection Fraction: a Community-Based Study. J. Am. Coll. Cardiol. 53 (13), 1119–1126. 10.1016/j.jacc.2008.11.051 19324256 PMC2736110

[B15] Li H. Duan Y. Chen B. Zhao Y. Su W. Wang S. (2020). New Pharmacological Treatments for Heart Failure with Reduced Ejection Fraction (HFrEF): A Bayesian Network Meta-Analysis. Medicine (Baltimore) 99 (5), e18341. 10.1097/MD.0000000000018341 32000355 PMC7004768

[B16] Massie B. M. Carson P. E. McMurray J. J. Komajda M. McKelvie R. Zile M. R. (2008). Irbesartan in Patients with Heart Failure and Preserved Ejection Fraction. N. Engl. J. Med. 359 (23), 2456–2467. 10.1056/NEJMoa0805450 19001508

[B17] Mozaffarian D. Benjamin E. J. Go A. S. Arnett D. K. Blaha M. J. Cushman M. (2015). Heart Disease and Stroke Statistics--2015 Update: a Report from the American Heart Association. Circulation 131 (4), e29–322. 10.1161/CIR.0000000000000152 25520374

[B18] Obokata M. Reddy Y. N. V. Pislaru S. V. Melenovsky V. Borlaug B. A. (2017). Evidence Supporting the Existence of a Distinct Obese Phenotype of Heart Failure with Preserved Ejection Fraction. Circulation 136 (1), 6–19. 10.1161/CIRCULATIONAHA.116.026807 28381470 PMC5501170

[B19] Pitt B. Pfeffer M. A. Assmann S. F. Boineau R. Anand I. S. Claggett B. (2014). Spironolactone for Heart Failure with Preserved Ejection Fraction. N. Engl. J. Med. 370 (15), 1383–1392. 10.1056/nejmoa1313731 24716680

[B20] Ponikowski P. Voors A. A. Anker S. D. Bueno H. Cleland J. G. Coats A. J. (20162016). 2016 ESC Guidelines for the Diagnosis and Treatment of Acute and Chronic Heart Failure: The Task Force for the Diagnosis and Treatment of Acute and Chronic Heart Failure of the European Society of Cardiology (ESC). Developed with the Special Contribution of the Heart Failure Association (HFA) of the ESC. Eur. J. Heart Fail. 18 (8), 891–975. 10.1002/ejhf.592 27207191

[B21] Ponikowski P. Voors A. A. Anker S. D. Bueno H. Cleland J. G. F. Coats A. J. S. (2016). 2016 ESC Guidelines for the Diagnosis and Treatment of Acute and Chronic Heart Failure: The Task Force for the Diagnosis and Treatment of Acute and Chronic Heart Failure of the European Society of Cardiology (ESC)Developed with the Special Contribution of the Heart Failure Association (HFA) of the ESC. Eur. Heart J. 37 (27), 2129–2200. 10.1093/eurheartj/ehw128 27206819

[B22] Redfield M. M. Chen H. H. Borlaug B. A. Semigran M. J. Lee K. L. Lewis G. (2013). Effect of Phosphodiesterase-5 Inhibition on Exercise Capacity and Clinical Status in Heart Failure with Preserved Ejection Fraction: a Randomized Clinical Trial. Jama 309 (12), 1268–1277. 10.1001/jama.2013.2024 23478662 PMC3835156

[B23] Redfield M. M. (2017). Heart Failure with Preserved Ejection Fraction. N. Engl. J. Med. 376 (9), 897. 10.1056/NEJMc1615918 28249128

[B24] Solomon S. D. McMurray J. J. V. Anand I. S. Ge J. Lam C. S. P. Maggioni A. P. (2019). Angiotensin-Neprilysin Inhibition in Heart Failure with Preserved Ejection Fraction. N. Engl. J. Med. 381 (17), 1609–1620. 10.1056/NEJMoa1908655 31475794

[B25] Solomon S. D. Zile M. Pieske B. Voors A. Shah A. Kraigher-Krainer E. (2012). The Angiotensin Receptor Neprilysin Inhibitor LCZ696 in Heart Failure with Preserved Ejection Fraction: a Phase 2 Double-Blind Randomised Controlled Trial. Lancet 380 (9851), 1387–1395. 10.1016/S0140-6736(12)61227-6 22932717

[B26] Yamamoto K. Origasa H. Hori M. (2013). Effects of Carvedilol on Heart Failure with Preserved Ejection Fraction: the Japanese Diastolic Heart Failure Study (J-DHF). Eur. J. Heart Fail. 15 (1), 110–118. 10.1093/eurjhf/hfs141 22983988

[B27] Yip G. W. Wang M. Wang T. Chan S. Fung J. W. Yeung L. (2008). The Hong Kong Diastolic Heart Failure Study: a Randomised Controlled Trial of Diuretics, Irbesartan and Ramipril on Quality of Life, Exercise Capacity, Left Ventricular Global and Regional Function in Heart Failure with a normal Ejection Fraction. Heart 94 (5), 573–580. 10.1136/hrt.2007.117978 18208835

[B28] Yusuf S. Pfeffer M. A. Swedberg K. Granger C. B. Held P. McMurray J. J. (2003). Effects of Candesartan in Patients with Chronic Heart Failure and Preserved Left-Ventricular Ejection Fraction: the CHARM-Preserved Trial. Lancet 362 (9386), 777–781. 10.1016/S0140-6736(03)14285-7 13678871

[B29] Zi M. Carmichael N. Lye M. (2003). The Effect of Quinapril on Functional Status of Elderly Patients with Diastolic Heart Failure. Cardiovasc. Drugs Ther. 17 (2), 133–139. 10.1023/a:1025387702212 12975595

[B30] Zile M. R. Baicu C. F. Gaasch W. H. (2004). Diastolic Heart Failure-Aabnormalities in Active Relaxation and Passive Stiffness of the Left Ventricle. N. Engl. J. Med. 350 (19), 1953–1959. 10.1056/NEJMoa032566 15128895

[B31] Zile M. R. O'Meara E. Claggett B. Prescott M. F. Solomon S. D. Swedberg K. (2019). Effects of Sacubitril/Valsartan on Biomarkers of Extracellular Matrix Regulation in Patients with HFrEF. J. Am. Coll. Cardiol. 73 (7), 795–806. 10.1016/j.jacc.2018.11.042 30784673

