# Supplementary material for: Comparison of Pharmacological Treatment Effects on Long-Time Outcomes in Heart Failure With Preserved Ejection Fraction: A Network Meta-analysis of Randomized Controlled Trials
Source: Front Pharmacol. 2021 Nov 24;12:707777. doi: 10.3389/fphar.2021.707777 (PMC8652335; doi:10.3389/fphar.2021.707777)
Supplement: Supplementary file 1 [file DataSheet4.pdf]

Confidence In Network Meta Analysis - CINeMA 2.0.0 - Project: **HFpEF network meta-analysis**

Heterogeneity

Define clinically important size of effect: Odds ratio 1

*Relative effect estimates below **1.000** and above **1.000** are considered clinically important.*
Importance of heterogeneity depends on the variability of effects in relation to a clinically important size of effect

The estimated value of between-study variance for the network meta-analysis is **0.000**

Comparison **ACEI:ARB**
Evidence: mixed

NMA estimate: **0.831**
95% intervals for NMA estimate
Confidence interval: **(0.433,1.594)**
Prediction interval: **(0.353,1.952)**

*Confidence and prediction intervals **agree** in relation to clinically important effect*

Heterogeneity judgment
No concerns ▼

Comparison **ACEI:Placebo**
Evidence: mixed

NMA estimate: **0.845**
95% intervals for NMA estimate
Confidence interval: **(0.448,1.595)**
Prediction interval: **(0.368,1.944)**

*Confidence and prediction intervals **agree** in relation to clinically important effect*

Heterogeneity judgment
No concerns ▼

Comparison **ARB:ARNI**
Evidence: mixed

NMA estimate: **1.036**
95% intervals for NMA estimate
Confidence interval: **(0.882,1.217)**
Prediction interval: **(0.839,1.279)**

*Confidence and prediction intervals **agree** in relation to clinically important effect*

Heterogeneity judgment
No concerns ▼

Comparison **ARB:Placebo**
Evidence: mixed

NMA estimate: **1.018**
95% intervals for NMA estimate
Confidence interval: **(0.877,1.180)**
Prediction interval: **(0.837,1.236)**

*Confidence and prediction intervals **agree** in relation to clinically important effect*

Heterogeneity judgment
No concerns ▼

Comparison Beta blockers:Placebo
Evidence: mixed

NMA estimate: **0.588**

95% intervals for NMA estimate

Confidence interval: **(0.363,0.953)**
Prediction interval: **(0.312,1.108)**

*Prediction interval extends into clinically important effects in **both** directions*

Heterogeneity judgment

Major concerns ▼

Comparison Digoxin:Placebo
Evidence: mixed

NMA estimate: **0.999**

95% intervals for NMA estimate

Confidence interval: **(0.744,1.342)**
Prediction interval: **(0.679,1.471)**

*Confidence and prediction intervals **agree** in relation to clinically important effect*

Heterogeneity judgment

No concerns ▼

Comparison MRA:Placebo
Evidence: mixed

NMA estimate: **0.910**

95% intervals for NMA estimate

Confidence interval: **(0.756,1.096)**
Prediction interval: **(0.714,1.161)**

*Confidence and prediction intervals **agree** in relation to clinically important effect*

Heterogeneity judgment

No concerns ▼

Comparison Placebo:Sildenafil
Evidence: mixed

NMA estimate: **0.153**

95% intervals for NMA estimate

Confidence interval: **(0.008,2.989)**
Prediction interval: **(0.003,7.552)**

*Confidence and prediction intervals **agree** in relation to clinically important effect*

Heterogeneity judgment

No concerns ▼

Comparison
Evidence: Placebo:Vericiguat 10mg mixed

NMA estimate: **0.452**

95% intervals for NMA estimate

Confidence interval: **(0.181,1.127)**
Prediction interval: **(0.136,1.499)**

*Confidence and prediction intervals **agree** in relation to clinically important effect*

Heterogeneity judgment

No concerns ▼

Comparison
Evidence: Placebo:Vericiguat 15mg mixed

NMA estimate: **0.697**

95% intervals for NMA estimate

Confidence interval: **(0.261,1.860)**
Prediction interval: **(0.192,2.526)**

*Confidence and prediction intervals **agree** in relation to clinically important effect*

Heterogeneity judgment

No concerns ▼

Comparison **Vericiguat 10mg:Vericiguat 15mg**
Evidence: mixed

NMA estimate: **1.542**
95% intervals for NMA estimate
Confidence interval: **(0.680,3.499)**
Prediction interval: **(0.527,4.516)**

*Confidence and prediction intervals **agree** in relation to clinically important effect*

Heterogeneity judgment
No concerns ▼

Comparison **ACEI:ARNI**
Evidence: indirect

NMA estimate: **0.861**
95% intervals for NMA estimate
Confidence interval: **(0.440,1.684)**
Prediction interval: **(0.357,2.075)**

*Confidence and prediction intervals **agree** in relation to clinically important effect*

Heterogeneity judgment
No concerns ▼

Comparison **ACEI:Beta blockers**
Evidence: indirect

NMA estimate: **1.437**
95% intervals for NMA estimate
Confidence interval: **(0.647,3.193)**
Prediction interval: **(0.505,4.094)**

*Confidence and prediction intervals **agree** in relation to clinically important effect*

Heterogeneity judgment
No concerns ▼

Comparison **ACEI:Digoxin**
Evidence: indirect

NMA estimate: **0.846**
95% intervals for NMA estimate
Confidence interval: **(0.420,1.703)**
Prediction interval: **(0.338,2.118)**

*Confidence and prediction intervals **agree** in relation to clinically important effect*

Heterogeneity judgment
No concerns ▼

Comparison **ACEI:MRA**
Evidence: indirect

NMA estimate: **0.929**
95% intervals for NMA estimate
Confidence interval: **(0.479,1.799)**
Prediction interval: **(0.390,2.211)**

*Confidence and prediction intervals **agree** in relation to clinically important effect*

Heterogeneity judgment
No concerns ▼

Comparison **ACEI:Sildenafil**
Evidence: indirect

NMA estimate: **0.129**
95% intervals for NMA estimate
Confidence interval: **(0.006,2.701)**
Prediction interval: **(0.002,6.969)**

*Confidence and prediction intervals **agree** in relation to clinically important effect*

Heterogeneity judgment
No concerns ▼

Comparison ACEI:Vericiguat 10mg
Evidence: indirect

NMA estimate: **0.382**

95% intervals for NMA estimate

Confidence interval: **(0.126,1.163)**

Prediction interval: **(0.089,1.645)**

*Confidence and prediction intervals **agree** in relation to clinically important effect*

Heterogeneity judgment

No concerns ▼

Comparison ACEI:Vericiguat 15mg
Evidence: indirect

NMA estimate: **0.589**

95% intervals for NMA estimate

Confidence interval: **(0.183,1.897)**

Prediction interval: **(0.127,2.730)**

*Confidence and prediction intervals **agree** in relation to clinically important effect*

Heterogeneity judgment

No concerns ▼

Comparison ARB:Beta blockers
Evidence: indirect

NMA estimate: **1.731**

95% intervals for NMA estimate

Confidence interval: **(1.044,2.869)**

Prediction interval: **(0.892,3.359)**

*Prediction interval extends into clinically important effects in **both** directions*

Heterogeneity judgment

Major concerns ▼

Comparison ARB:Digoxin
Evidence: indirect

NMA estimate: **1.018**

95% intervals for NMA estimate

Confidence interval: **(0.732,1.416)**

Prediction interval: **(0.661,1.570)**

*Confidence and prediction intervals **agree** in relation to clinically important effect*

Heterogeneity judgment

No concerns ▼

Comparison ARB:MRA
Evidence: indirect

NMA estimate: **1.118**

95% intervals for NMA estimate

Confidence interval: **(0.882,1.418)**

Prediction interval: **(0.819,1.527)**

*Confidence and prediction intervals **agree** in relation to clinically important effect*

Heterogeneity judgment

No concerns ▼

Comparison ARB:Sildenafil
Evidence: indirect

NMA estimate: **0.155**

95% intervals for NMA estimate

Confidence interval: **(0.008,3.052)**

Prediction interval: **(0.003,7.721)**

*Confidence and prediction intervals **agree** in relation to clinically important effect*

Heterogeneity judgment

No concerns ▼

Comparison ARB:Vericiguat 10mg
Evidence: indirect

NMA estimate: **0.460**
95% intervals for NMA estimate
Confidence interval: **(0.182,1.161)**
Prediction interval: **(0.137,1.549)**

*Confidence and prediction intervals **agree** in relation to clinically important effect*

Heterogeneity judgment
No concerns ▼

Comparison ARB:Vericiguat 15mg
Evidence: indirect

NMA estimate: **0.709**
95% intervals for NMA estimate
Confidence interval: **(0.263,1.914)**
Prediction interval: **(0.193,2.608)**

*Confidence and prediction intervals **agree** in relation to clinically important effect*

Heterogeneity judgment
No concerns ▼

Comparison ARNI:Beta blockers
Evidence: indirect

NMA estimate: **1.670**
95% intervals for NMA estimate
Confidence interval: **(0.983,2.840)**
Prediction interval: **(0.833,3.350)**

*Confidence and prediction intervals **agree** in relation to clinically important effect*

Heterogeneity judgment
No concerns ▼

Comparison ARNI:Digoxin
Evidence: indirect

NMA estimate: **0.983**
95% intervals for NMA estimate
Confidence interval: **(0.681,1.419)**
Prediction interval: **(0.607,1.591)**

*Confidence and prediction intervals **agree** in relation to clinically important effect*

Heterogeneity judgment
No concerns ▼

Comparison ARNI:MRA
Evidence: indirect

NMA estimate: **1.079**
95% intervals for NMA estimate
Confidence interval: **(0.810,1.438)**
Prediction interval: **(0.741,1.572)**

*Confidence and prediction intervals **agree** in relation to clinically important effect*

Heterogeneity judgment
No concerns ▼

Comparison ARNI:Placebo
Evidence: indirect

NMA estimate: **0.982**
95% intervals for NMA estimate
Confidence interval: **(0.789,1.223)**
Prediction interval: **(0.737,1.309)**

*Confidence and prediction intervals **agree** in relation to clinically important effect*

Heterogeneity judgment
No concerns ▼

Comparison ARNI:Sildenafil
Evidence: indirect

NMA estimate: **0.150**

95% intervals for NMA estimate

Confidence interval: **(0.008,2.959)**
Prediction interval: **(0.003,7.495)**

Confidence and prediction intervals ***agree*** *in relation to clinically important effect*

Heterogeneity judgment
No concerns ▼

Comparison ARNI:Vericiguat 10mg
Evidence: indirect

NMA estimate: **0.444**

95% intervals for NMA estimate

Confidence interval: **(0.173,1.136)**
Prediction interval: **(0.129,1.523)**

Confidence and prediction intervals ***agree*** *in relation to clinically important effect*

Heterogeneity judgment
No concerns ▼

Comparison ARNI:Vericiguat 15mg
Evidence: indirect

NMA estimate: **0.685**

95% intervals for NMA estimate

Confidence interval: **(0.251,1.872)**
Prediction interval: **(0.183,2.560)**

Confidence and prediction intervals ***agree*** *in relation to clinically important effect*

Heterogeneity judgment
No concerns ▼

Comparison Beta blockers:Digoxin
Evidence: indirect

NMA estimate: **0.588**

95% intervals for NMA estimate

Confidence interval: **(0.334,1.036)**
Prediction interval: **(0.280,1.236)**

Confidence and prediction intervals ***agree*** *in relation to clinically important effect*

Heterogeneity judgment
No concerns ▼

Comparison Beta blockers:MRA
Evidence: indirect

NMA estimate: **0.646**

95% intervals for NMA estimate

Confidence interval: **(0.385,1.084)**
Prediction interval: **(0.328,1.274)**

Confidence and prediction intervals ***agree*** *in relation to clinically important effect*

Heterogeneity judgment
No concerns ▼

Comparison Beta blockers:Sildenafil
Evidence: indirect

NMA estimate: **0.090**

95% intervals for NMA estimate

Confidence interval: **(0.004,1.827)**
Prediction interval: **(0.002,4.673)**

Confidence and prediction intervals ***agree*** *in relation to clinically important effect*

Heterogeneity judgment
No concerns ▼

Comparison **Beta blockers:Vericiguat 10mg**
Evidence: indirect

NMA estimate: **0.266**
95% intervals for NMA estimate
Confidence interval: **(0.095,0.747)**
Prediction interval: **(0.068,1.031)**

*Prediction interval extends into clinically important effects in **both** directions*

Heterogeneity judgment
Major concerns ▼

Comparison **Beta blockers:Vericiguat 15mg**
Evidence: indirect

NMA estimate: **0.410**
95% intervals for NMA estimate
Confidence interval: **(0.137,1.224)**
Prediction interval: **(0.098,1.721)**

*Confidence and prediction intervals **agree** in relation to clinically important effect*

Heterogeneity judgment
No concerns ▼

Comparison **Digoxin:MRA**
Evidence: indirect

NMA estimate: **1.098**
95% intervals for NMA estimate
Confidence interval: **(0.775,1.555)**
Prediction interval: **(0.695,1.733)**

*Confidence and prediction intervals **agree** in relation to clinically important effect*

Heterogeneity judgment
No concerns ▼

Comparison **Digoxin:Sildenafil**
Evidence: indirect

NMA estimate: **0.152**
95% intervals for NMA estimate
Confidence interval: **(0.008,3.030)**
Prediction interval: **(0.003,7.691)**

*Confidence and prediction intervals **agree** in relation to clinically important effect*

Heterogeneity judgment
No concerns ▼

Comparison **Digoxin:Vericiguat 10mg**
Evidence: indirect

NMA estimate: **0.452**
95% intervals for NMA estimate
Confidence interval: **(0.173,1.180)**
Prediction interval: **(0.128,1.592)**

*Confidence and prediction intervals **agree** in relation to clinically important effect*

Heterogeneity judgment
No concerns ▼

Comparison **Digoxin:Vericiguat 15mg**
Evidence: indirect

NMA estimate: **0.697**
95% intervals for NMA estimate
Confidence interval: **(0.250,1.941)**
Prediction interval: **(0.182,2.671)**

*Confidence and prediction intervals **agree** in relation to clinically important effect*

Heterogeneity judgment
No concerns ▼

Comparison MRA:Sildenafil
Evidence: indirect

NMA estimate: **0.139**
95% intervals for NMA estimate
Confidence interval: **(0.007,2.736)**
Prediction interval: **(0.003,6.925)**

*Confidence and prediction intervals **agree** in relation to clinically important effect*

Heterogeneity judgment
No concerns ▼

Comparison MRA:Vericiguat 10mg
Evidence: indirect

NMA estimate: **0.411**
95% intervals for NMA estimate
Confidence interval: **(0.162,1.046)**
Prediction interval: **(0.121,1.398)**

*Confidence and prediction intervals **agree** in relation to clinically important effect*

Heterogeneity judgment
No concerns ▼

Comparison MRA:Vericiguat 15mg
Evidence: indirect

NMA estimate: **0.635**
95% intervals for NMA estimate
Confidence interval: **(0.234,1.723)**
Prediction interval: **(0.171,2.352)**

*Confidence and prediction intervals **agree** in relation to clinically important effect*

Heterogeneity judgment
No concerns ▼

Comparison Sildenafil:Vericiguat 10mg
Evidence: indirect

NMA estimate: **2.964**
95% intervals for NMA estimate
Confidence interval: **(0.132,66.620)**
Prediction interval: **(0.050,175.686)**

*Confidence and prediction intervals **agree** in relation to clinically important effect*

Heterogeneity judgment
No concerns ▼

Comparison Sildenafil:Vericiguat 15mg
Evidence: indirect

NMA estimate: **4.572**
95% intervals for NMA estimate
Confidence interval: **(0.199,104.878)**
Prediction interval: **(0.075,278.356)**

*Confidence and prediction intervals **agree** in relation to clinically important effect*

Heterogeneity judgment
No concerns ▼
