# Supplementary material for: Comparison of Pharmacological Treatment Effects on Long-Time Outcomes in Heart Failure With Preserved Ejection Fraction: A Network Meta-analysis of Randomized Controlled Trials
Source: Front Pharmacol. 2021 Nov 24;12:707777. doi: 10.3389/fphar.2021.707777 (PMC8652335; doi:10.3389/fphar.2021.707777)
Supplement: Supplementary file 2 [file DataSheet1.doc]

**Supplementary Table 1 A. Final search strategy for PubMed**

| **#ID** | **Topic or intervention** | **Query** | **Records** |
| --- | --- | --- | --- |
| **#1** | Disease | (“heart failure with preserved ejection fraction”[Mesh]) OR (“diastolic heart failure”[Mesh] ) OR (“heart failure with preserved ejection fraction”[Title/Abstract] ) OR (“diastolic heart failure”[Title/Abstract]) | 4,466 |
| **#2** | Study design | (“randomized controlled trial”[Publication Type] OR (randomized[Publication Typet]) OR “randomized controlled trial”[Title/Abstract] OR randomization [Title/Abstract] | 1,275,746 |
| **#3** | angiotensin receptor neprilysin inhibitor | (“angiotensin receptor neprilysin inhibitor”[Mesh]) OR (“sacubitril–valsarta” [Mesh] ) OR (“LCZ696” [Mesh]) OR (“angiotensin receptor neprilysin inhibitor” [Title/Abstract]) OR (“sacubitril–valsarta”[Title/Abstract]) OR (“LCZ696” [Title/Abstract] ) | 512 |
| **#4** | angiotensin converting enzyme inhibitors | (“angiotensin converting enzyme inhibitor”[Mesh]) OR (“perindopril” [Mesh] ) OR (“quinapril” [Mesh] ) OR (“perindopri” [Mesh] ) OR (“benazepril” [Mesh] ) OR (“catopril” [Mesh] ) OR (“fosinopril” [Mesh] ) OR (“enalapril” [Mesh] ) OR (“ramipril” [Mesh] ) OR (“angiotensin converting enzyme inhibitor” [Title/Abstract]) OR (“perindopril” [Title/Abstract]) OR (“quinapril” [Title/Abstract] ) OR (“perindopri” [Title/Abstract] ) OR (“benazepril” [Title/Abstract] ) OR (“catopril” [Title/Abstract] ) OR (“Fosinopril” [Title/Abstract] ) OR (“Enalapril” [Title/Abstract] ) OR (“ramipril” [Title/Abstract] ) | 20,812 |
| **#5** | angiotensin receptor blockers | (“angiotensin receptor blockers”[Mesh]) OR (“irbesartan” [Mesh] ) OR (“valsartan” [Mesh] ) OR (“candesartan” [Mesh]) OR (“losartan” [Mesh]) OR (“angiotensin receptor blockers”[Title/Abstract]) OR (“Irbesartan” [Title/Abstract] ) OR (“valsartan” [Title/Abstract]) OR (“candesartan” [Title/Abstract]) OR (“losartan” [Title/Abstract] ) | 21,788 |
| **#6** | beta blockers | (“beta blockers”[Mesh]) OR (“propranolol” [Mesh] ) OR (“carvedilol” [Mesh] ) OR (“metoprolol” [Mesh]) OR (“bisoprolol” [Mesh]) OR (“beta blockers”[Title/Abstract]) OR (“propranolol”[Title/Abstract]) OR (“carvedilol”[Title/Abstract]) OR (“metoprolol” [Title/Abstract] ) OR (“bisoprolol” [Title/Abstract] ) | 74,593 |
| **#7** | mineralocorticoid receptor antagonists | (“mineralocorticoid receptor antagonists”[Mesh]) OR (“spirolactone” [Mesh] ) OR (“eplerenone” [Mesh] ) OR (“antisterone” [Mesh]) OR (“aldactone” [Mesh]) OR “mineralocorticoid receptor antagonists”[Title/Abstract]) OR (“spirolactone” [Title/Abstract]) OR (“eplerenone”[Title/Abstract]) OR (“antisterone” [Title/Abstract] ) OR (“aldactone” [Title/Abstract] ) | 6,639 |
| **#8** | digoxin | (“digoxin”[Mesh]) OR (“digoxin” [Title/Abstract] ) | 16,534 |
| **#9** | phosphodiesterase-5 inhibition or sidenafi | (“phosphodiesterase-5 inhibition”[Mesh]) OR (“sidenafi” [Mesh]) OR (“tadalafil” [Mesh]) OR (“phosphodiesterase-5 inhibition”[Title/Abstract]) OR (“sidenafi” [Title/Abstract]) OR (“tadalafil” [Title/Abstract] ) | 2,489 |
| **#10** | soluble guanylate cyclase stimulator | (“soluble guanylate cyclase stimulator”[Mesh]) OR (“vericiguat” [Mesh]) OR (“soluble guanylate cyclase stimulator”[Title/Abstract]) OR (“vericiguat” [Title/Abstract]) | 206 |
| **#11** | sodium-glucose cotransporter-2 | (“sodium-glucose cotransporter-2”[Mesh]) OR (“dapagliflozin”[Mesh]) OR (“ canagliflozin” [Mesh] ) OR (“sodium-glucose cotransporter-2”[Title/Abstract]) OR (“dapagliflozin” [Title/Abstract] ) OR (“ canagliflozin” [Title/Abstract] ) | 4,053 |
| **#12** | diuretic | (“loop diuretic ”[Mesh]) OR (“furosemide” [Mesh] ) OR (“bumetanide” [Mesh] ) OR (“torasemide” [Mesh]) OR (“azosemide” [Mesh]) OR (“eplerenone” [Mesh]) OR (“hydroclorotiazides” [Mesh]) OR (“loop diuretic ”[Title/Abstract]) OR (“furosemide”[Title/Abstract]) OR (“bumetanide”[Title/Abstract]) OR (“torasemide” [Title/Abstract]) OR (“azosemide” [Title/Abstract]) OR (“eplerenone” [Title/Abstract]) OR (“hydroclorotiazides” [Title/Abstract]) | 20,349 |
| **#13** | Final query | #1 AND #2 AND (#3 OR #4 OR #5 OR #6 OR #7 OR #8 OR #9 OR #10 OR #11 OR #12) | 377 |

**Supplementary Table 1 B. Final search strategy for Cochrane Central Register of Controlled Trials**

| **#ID** | **Topic or intervention** | **Query** | **Records** |
| --- | --- | --- | --- |
| **#1** | Disease | (“heart failure with preserved ejection fraction”[Mesh]) OR (“diastolic heart failure”[Mesh] ) OR (“heart failure with preserved ejection fraction”[Title/Abstract] ) OR (“diastolic heart failure”[Title/Abstract]) | 5,717 |
| **#2** | Study design | (“randomized controlled trial”[Publication Type] OR (randomized[Publication Typet]) OR “randomized controlled trial”[Title/Abstract] OR randomization [Title/Abstract] | 1,087,016 |
| **#3** | angiotensin receptor neprilysin inhibitor | (“angiotensin receptor neprilysin inhibitor”[Mesh]) OR (“sacubitril–valsarta” [Mesh] ) OR (“LCZ696” [Mesh]) OR (“angiotensin receptor neprilysin inhibitor” [Title/Abstract]) OR (“sacubitril–valsarta”[Title/Abstract]) OR (“LCZ696” [Title/Abstract] ) | 455 |
| **#4** | angiotensin converting enzyme inhibitors | (“angiotensin converting enzyme inhibitor”[Mesh]) OR (“perindopril” [Mesh] ) OR (“quinapril” [Mesh] ) OR (“perindopri” [Mesh] ) OR (“benazepril” [Mesh] ) OR (“catopril” [Mesh] ) OR (“fosinopril” [Mesh] ) OR (“enalapril” [Mesh] ) OR (“ramipril” [Mesh] ) OR (“angiotensin converting enzyme inhibitor” [Title/Abstract]) OR (“perindopril” [Title/Abstract]) OR (“quinapril” [Title/Abstract] ) OR (“perindopri” [Title/Abstract] ) OR (“benazepril” [Title/Abstract] ) OR (“catopril” [Title/Abstract] ) OR (“Fosinopril” [Title/Abstract] ) OR (“Enalapril” [Title/Abstract] ) OR (“ramipril” [Title/Abstract] ) | 6,253 |
| **#5** | angiotensin receptor blockers | (“angiotensin receptor blockers”[Mesh]) OR (“irbesartan” [Mesh] ) OR (“valsartan” [Mesh] ) OR (“candesartan” [Mesh]) OR (“losartan” [Mesh]) OR (“angiotensin receptor blockers”[Title/Abstract]) OR (“Irbesartan” [Title/Abstract] ) OR (“valsartan” [Title/Abstract]) OR (“candesartan” [Title/Abstract]) OR (“losartan” [Title/Abstract] ) | 5,194 |
| **#6** | beta blockers | (“beta blockers”[Mesh]) OR (“propranolol” [Mesh] ) OR (“carvedilol” [Mesh] ) OR (“metoprolol” [Mesh]) OR (“bisoprolol” [Mesh]) OR (“beta blockers”[Title/Abstract]) OR (“propranolol”[Title/Abstract]) OR (“carvedilol”[Title/Abstract]) OR (“metoprolol” [Title/Abstract] ) OR (“bisoprolol” [Title/Abstract] ) | 5,947 |
| **#7** | mineralocorticoid receptor antagonists | (“mineralocorticoid receptor antagonists”[Mesh]) OR (“spirolactone” [Mesh] ) OR (“eplerenone” [Mesh] ) OR (“antisterone” [Mesh]) OR (“aldactone” [Mesh]) OR “mineralocorticoid receptor antagonists”[Title/Abstract]) OR (“spirolactone” [Title/Abstract]) OR (“eplerenone”[Title/Abstract]) OR (“antisterone” [Title/Abstract] ) OR (“aldactone” [Title/Abstract] ) | 1,322 |
| **#8** | digoxin | (“digoxin”[Mesh]) OR (“digoxin” [Title/Abstract] ) | 1,980 |
| **#9** | phosphodiesterase-5 inhibition or sidenafi | (“phosphodiesterase-5 inhibition”[Mesh]) OR (“sidenafi” [Mesh]) OR (“tadalafil” [Mesh]) OR (“phosphodiesterase-5 inhibition”[Title/Abstract]) OR (“sidenafi” [Title/Abstract]) OR (“tadalafil” [Title/Abstract] ) | 130 |
| **#10** | soluble guanylate cyclase stimulator | (“soluble guanylate cyclase stimulator”[Mesh]) OR (“vericiguat” [Mesh]) OR (“soluble guanylate cyclase stimulator”[Title/Abstract]) OR (“vericiguat” [Title/Abstract]) | 154 |
| **#11** | sodium-glucose cotransporter-2 | (“sodium-glucose cotransporter-2”[Mesh]) OR (“dapagliflozin”[Mesh]) OR (“ canagliflozin” [Mesh] ) OR (“sodium-glucose cotransporter-2”[Title/Abstract]) OR (“dapagliflozin” [Title/Abstract] ) OR (“ canagliflozin” [Title/Abstract] ) | 2,953 |
| **#12** | diuretic | (“loop diuretic ”[Mesh]) OR (“furosemide” [Mesh] ) OR (“bumetanide” [Mesh] ) OR (“torasemide” [Mesh]) OR (“azosemide” [Mesh]) OR (“eplerenone” [Mesh]) OR (“hydroclorotiazides” [Mesh]) OR (“loop diuretic ”[Title/Abstract]) OR (“furosemide”[Title/Abstract]) OR (“bumetanide”[Title/Abstract]) OR (“torasemide” [Title/Abstract]) OR (“azosemide” [Title/Abstract]) OR (“eplerenone” [Title/Abstract]) OR (“hydroclorotiazides” [Title/Abstract]) | 3,358 |
| **#12** | Final query | #1 AND #2 AND (#3 OR #4 OR #5 OR #6 OR #7 OR #8 OR #9 OR #10 OR #11 AND #12 ) | 293 |
