# Supplementary material for: Comparison of Pharmacological Treatment Effects on Long-Time Outcomes in Heart Failure With Preserved Ejection Fraction: A Network Meta-analysis of Randomized Controlled Trials
Source: Front Pharmacol. 2021 Nov 24;12:707777. doi: 10.3389/fphar.2021.707777 (PMC8652335; doi:10.3389/fphar.2021.707777)
Supplement: Supplementary file 3 [file DataSheet3.pdf]

Confidence In Network Meta Analysis - CINeMA 2.0.0

Incoherence

Define clinically important size of effect: Odds ratio 1

*Relative effect estimates below **1.000** and above **1.000** are considered clinically important. Importance of Incoherence depends on the variability of direct and indirect effects in relation to a clinically important size of effect*

Global test based on a random-effects design-by-treatment interaction model

χ^2 statistic: 1.912 (2 degrees of freedom), P value: 0.384

Local tests: Separating indirect from direct evidence

**Comparison**	**ACEI:ARB**
**Evidence: mixed**	
NMA odds ratio:	**0.831(0.433,1.594)**
Direct odds ratio:	**0.443(0.018,11.167)**
Indirect odds ratio:	**0.853(0.439,1.659)**
**Inconsistency measures**	
Ratio of odds ratios:	**0.519(0.019,14.009)**
P value:	**0.697**
Incoherence judgment	No concerns ▼

**Comparison**	**ACEI:Placebo**
**Evidence: mixed**	
NMA odds ratio:	**0.845(0.448,1.595)**
Direct odds ratio:	**0.835(0.442,1.577)**
Indirect odds ratio:	**407.198(0.000,834213564.430)**
**Inconsistency measures**	
Ratio of odds ratios:	**0.002(0.000,4261.174)**
P value:	**0.404**
Incoherence judgment	No concerns ▼

**Comparison**	**ARB:ARNI**
**Evidence: direct**	
Direct odds ratio:	**1.036(0.882,1.217)**
**Inconsistency measures:** Not applicable	
Incoherence judgment	No concerns ▼

**Comparison**	**ARB:Placebo**
**Evidence: direct**	
Direct odds ratio:	**1.016(0.876,1.179)**
**Inconsistency measures:** Not applicable	
Incoherence judgment	No concerns ▼

**Comparison**	**Beta blockers:Placebo**
**Evidence: direct**	
Direct odds ratio:	**0.588(0.363,0.953)**
**Inconsistency measures:** Not applicable	
Incoherence judgment	No concerns ▼

**Comparison**	**Digoxin:Placebo**
**Evidence: direct**	
Direct odds ratio:	**0.999(0.744,1.342)**
**Inconsistency measures:** Not applicable	
Incoherence judgment	No concerns ▼

**Comparison**	**MRA:Placebo**
**Evidence: direct**	
Direct odds ratio:	**0.910(0.756,1.096)**
**Inconsistency measures:** Not applicable	
Incoherence judgment	No concerns ▼

**Comparison**	**Placebo:Sildenafil**
**Evidence: direct**	
Direct odds ratio:	**0.153(0.008,2.989)**
**Inconsistency measures:** Not applicable	
Incoherence judgment	No concerns ▼

**Comparison**	**Placebo:Vericiguat 10mg**
**Evidence: direct**	
Direct odds ratio:	**0.452(0.181,1.127)**
**Inconsistency measures:** Not applicable	
Incoherence judgment	No concerns ▼

**Comparison**	**Placebo:Vericiguat 15mg**
**Evidence: direct**	
Direct odds ratio:	**0.697(0.261,1.860)**
**Inconsistency measures:** Not applicable	
Incoherence judgment	No concerns ▼

**Comparison**	**Vericiguat 10mg:Vericiguat 15mg**
**Evidence: direct**	
Direct odds ratio:	**1.542(0.680,3.499)**
**Inconsistency measures:** Not applicable	
Incoherence judgment	No concerns ▼

Comparison	ACEI:ARNI
**Evidence: indirect**	
Indirect odds ratio:	**0.861(0.440,1.684)**
**Inconsistency measures:** Not applicable	
Incoherence judgment	No concerns ▼

Comparison	ACEI:Beta blockers
**Evidence: indirect**	
Indirect odds ratio:	**1.437(0.647,3.193)**
**Inconsistency measures:** Not applicable	
Incoherence judgment	No concerns ▼

Comparison	ACEI:Digoxin
**Evidence: indirect**	
Indirect odds ratio:	**0.846(0.420,1.703)**
**Inconsistency measures:** Not applicable	
Incoherence judgment	No concerns ▼

Comparison	ACEI:MRA
**Evidence: indirect**	
Indirect odds ratio:	**0.929(0.479,1.799)**
**Inconsistency measures:** Not applicable	
Incoherence judgment	No concerns ▼

Comparison	ACEI:Sildenafil
**Evidence: indirect**	
Indirect odds ratio:	**0.129(0.006,2.701)**
**Inconsistency measures:** Not applicable	
Incoherence judgment	No concerns ▼

Comparison	ACEI:Vericiguat 10mg
**Evidence: indirect**	
Indirect odds ratio:	**0.382(0.126,1.163)**
**Inconsistency measures:** Not applicable	
Incoherence judgment	No concerns ▼

Comparison	ACEI:Vericiguat 15mg
**Evidence: indirect**	
Indirect odds ratio:	**0.589(0.183,1.897)**
**Inconsistency measures:** Not applicable	
Incoherence judgment	No concerns ▼

Comparison	ARB:Beta blockers
**Evidence: indirect**	
Indirect odds ratio:	**1.731(1.044,2.869)**
**Inconsistency measures:** Not applicable	
Incoherence judgment	No concerns ▼

Comparison	ARB:Digoxin
**Evidence: indirect**	
Indirect odds ratio:	**1.018(0.732,1.416)**
**Inconsistency measures:** Not applicable	
Incoherence judgment	No concerns ▼

Comparison	ARB:MRA
**Evidence: indirect**	
Indirect odds ratio:	**1.118(0.882,1.418)**
**Inconsistency measures:** Not applicable	
Incoherence judgment	No concerns ▼

Comparison	ARB:Sildenafil
**Evidence: indirect**	
Indirect odds ratio:	**0.155(0.008,3.052)**
**Inconsistency measures:** Not applicable	
Incoherence judgment	No concerns ▼

Comparison	ARB:Vericiguat 10mg
**Evidence: indirect**	
Indirect odds ratio:	**0.460(0.182,1.161)**
**Inconsistency measures:** Not applicable	
Incoherence judgment	No concerns ▼

Comparison	ARB:Vericiguat 15mg
**Evidence: indirect**	
Indirect odds ratio:	**0.709(0.263,1.914)**
**Inconsistency measures:** Not applicable	
Incoherence judgment	No concerns ▼

Comparison	ARNI:Beta blockers
**Evidence: indirect**	
Indirect odds ratio:	**1.670(0.983,2.840)**
**Inconsistency measures:** Not applicable	
Incoherence judgment	No concerns ▼

Comparison	ARNI:Digoxin
**Evidence: indirect**	
Indirect odds ratio:	**0.983(0.681,1.419)**
**Inconsistency measures:** Not applicable	
Incoherence judgment	No concerns ▼

Comparison	ARNI:MRA
**Evidence: indirect**	
Indirect odds ratio:	**1.079(0.810,1.438)**
**Inconsistency measures:** Not applicable	
Incoherence judgment	No concerns ▼

Comparison **ARNI:Placebo**
Evidence: indirect

Indirect odds ratio: **0.982(0.789,1.223)**
Inconsistency measures: Not applicable

Incoherence judgment No concerns ▼

Comparison **ARNI:Sildenafil**
Evidence: indirect

Indirect odds ratio: **0.150(0.008,2.959)**
Inconsistency measures: Not applicable

Incoherence judgment No concerns ▼

Comparison **ARNI:Vericiguat 10mg**
Evidence: indirect

Indirect odds ratio: **0.444(0.173,1.136)**
Inconsistency measures: Not applicable

Incoherence judgment No concerns ▼

Comparison **ARNI:Vericiguat 15mg**
Evidence: indirect

Indirect odds ratio: **0.685(0.251,1.872)**
Inconsistency measures: Not applicable

Incoherence judgment No concerns ▼

Comparison **Beta blockers:Digoxin**
Evidence: indirect

Indirect odds ratio: **0.588(0.334,1.036)**
Inconsistency measures: Not applicable

Incoherence judgment No concerns ▼

Comparison **Beta blockers:MRA**
Evidence: indirect

Indirect odds ratio: **0.646(0.385,1.084)**
Inconsistency measures: Not applicable

Incoherence judgment No concerns ▼

Comparison **Beta blockers:Sildenafil**
Evidence: indirect

Indirect odds ratio: **0.090(0.004,1.827)**
Inconsistency measures: Not applicable

Incoherence judgment No concerns ▼

Comparison **Beta blockers:Vericiguat 10mg**
Evidence: indirect

Indirect odds ratio: **0.266(0.095,0.747)**
Inconsistency measures: Not applicable

Incoherence judgment No concerns ▼

Comparison **Beta blockers:Vericiguat 15mg**
Evidence: indirect

Indirect odds ratio: **0.410(0.137,1.224)**
Inconsistency measures: Not applicable

Incoherence judgment No concerns ▼

Comparison **Digoxin:MRA**
Evidence: indirect

Indirect odds ratio: **1.098(0.775,1.555)**
Inconsistency measures: Not applicable

Incoherence judgment No concerns ▼

Comparison **Digoxin:Sildenafil**
Evidence: indirect

Indirect odds ratio: **0.152(0.008,3.030)**
Inconsistency measures: Not applicable

Incoherence judgment No concerns ▼

Comparison **Digoxin:Vericiguat 10mg**
Evidence: indirect

Indirect odds ratio: **0.452(0.173,1.180)**
Inconsistency measures: Not applicable

Incoherence judgment No concerns ▼

Comparison **Digoxin:Vericiguat 15mg**
Evidence: indirect

Indirect odds ratio: **0.697(0.250,1.941)**
Inconsistency measures: Not applicable

Incoherence judgment No concerns ▼

Comparison **MRA:Sildenafil**
Evidence: indirect

Indirect odds ratio: **0.139(0.007,2.736)**
Inconsistency measures: Not applicable

Incoherence judgment No concerns ▼

Comparison **MRA:Vericiguat 10mg**
Evidence: indirect

Indirect odds ratio: **0.411(0.162,1.046)**
Inconsistency measures: Not applicable

Incoherence judgment No concerns ▼

Comparison **MRA:Vericiguat 15mg**
Evidence: indirect

Indirect odds ratio: **0.635(0.234,1.723)**
Inconsistency measures: Not applicable

Incoherence judgment No concerns ▼

Comparison **Sildenafil:Vericiguat 10mg**
Evidence: indirect

Indirect odds ratio: **2.964(0.132,66.620)**
Inconsistency measures: Not applicable

Incoherence judgment No concerns ▼

Comparison **Sildenafil:Vericiguat 15mg**
Evidence: indirect

Indirect odds ratio: **4.572(0.199,104.878)**
Inconsistency measures: Not applicable

Incoherence judgment No concerns ▼
