# Supplementary material for: Comparison of Pharmacological Treatment Effects on Long-Time Outcomes in Heart Failure With Preserved Ejection Fraction: A Network Meta-analysis of Randomized Controlled Trials
Source: Front Pharmacol. 2021 Nov 24;12:707777. doi: 10.3389/fphar.2021.707777 (PMC8652335; doi:10.3389/fphar.2021.707777)
Supplement: Supplementary file 4 [file DataSheet5.pdf]

Confidence In Network Meta Analysis - CINeMA 2.0.0 - Project: **HFpEF network meta-analysis**

HFpEF network meta-analysis

Comparison	Number of Studies	Within-study bias	Reporting bias	Indirectness	Imprecision	Heterogeneity	Incoherence	Confidence rating	Reason(s) f downgradi
				Mixed evidence					
**ACEI vs ARB**	1	--	--	No concerns	Major concerns	No concerns	No concerns	Low	|
**ACEI vs Placebo**	3	--	--	No concerns	Major concerns	No concerns	No concerns	Low	|
**ARB vs ARNI**	2	--	--	Some concerns	Major concerns	No concerns	No concerns	Low	|
**ARB vs Placebo**	2	--	--	No concerns	Major concerns	No concerns	No concerns	Low	|
**Beta blockers vs Placebo**	2	--	--	No concerns	No concerns	Major concerns	No concerns	Low	|
**Digoxin vs Placebo**	1	--	--	No concerns	Major concerns	No concerns	No concerns	Low	|
**MRA vs Placebo**	2	--	--	No concerns	Major concerns	No concerns	No concerns	Low	|
**Placebo vs Sildenafil**	1	--	--	No concerns	Major concerns	No concerns	No concerns	Low	|
**Placebo vs Vericiguat 10mg**	1	--	--	No concerns	Major concerns	No concerns	No concerns	Low	|
**Placebo vs Vericiguat 15mg**	1	--	--	No concerns	Major concerns	No concerns	No concerns	Low	|
**Vericiguat 10mg vs Vericiguat 15mg**	1	--	--	No concerns	Major concerns	No concerns	No concerns	Moderate	|
				Indirect evidence					
**ACEI vs ARNI**	--	--	--	No concerns	Major concerns	No concerns	No concerns	Low	|
**ACEI vs Beta blockers**	--	--	--	No concerns	Major concerns	No concerns	No concerns	Moderate	|
**ACEI vs Digoxin**	--	--	--	No concerns	Major concerns	No concerns	No concerns	Moderate	|
**ACEI vs MRA**	--	--	--	No concerns	Major concerns	No concerns	No concerns	Moderate	|
**ACEI vs Sildenafil**	--	--	--	No concerns	Major concerns	No concerns	No concerns	Moderate	|

Comparison	Number of Studies	Within-study bias	Reporting bias	Indirectness	Imprecision	Heterogeneity	Incoherence	Confidence rating	Reason(s) f downgradi
ACEI vs Vericiguat 10mg	--	--	--	No concerns	Major concerns	No concerns	No concerns	Moderate	
ACEI vs Vericiguat 15mg	--	--	--	No concerns	Major concerns	No concerns	No concerns	Moderate	
ARB vs Beta blockers	--	--	--	No concerns	No concerns	Major concerns	No concerns	Moderate	
ARB vs Digoxin	--	--	--	No concerns	Major concerns	No concerns	No concerns	Moderate	
ARB vs MRA	--	--	--	No concerns	Major concerns	No concerns	No concerns	Moderate	
ARB vs Sildenafil	--	--	--	No concerns	Major concerns	No concerns	No concerns	Moderate	
ARB vs Vericiguat 10mg	--	--	--	No concerns	Major concerns	No concerns	No concerns	Moderate	
ARB vs Vericiguat 15mg	--	--	--	No concerns	Major concerns	No concerns	No concerns	Moderate	
ARNI vs Beta blockers	--	--	--	No concerns	Major concerns	No concerns	No concerns	Moderate	
ARNI vs Digoxin	--	--	--	No concerns	Major concerns	No concerns	No concerns	Moderate	
ARNI vs MRA	--	--	--	No concerns	Major concerns	No concerns	No concerns	Moderate	
ARNI vs Placebo	--	--	--	No concerns	Major concerns	No concerns	No concerns	Moderate	
ARNI vs Sildenafil	--	--	--	No concerns	Major concerns	No concerns	No concerns	Moderate	
ARNI vs Vericiguat 10mg	--	--	--	No concerns	Major concerns	No concerns	No concerns	Moderate	
ARNI vs Vericiguat 15mg	--	--	--	No concerns	Major concerns	No concerns	No concerns	Moderate	
Beta blockers vs Digoxin	--	--	--	No concerns	Major concerns	No concerns	No concerns	Moderate	
Beta blockers vs MRA	--	--	--	No concerns	Major concerns	No concerns	No concerns	Moderate	
Beta blockers vs Sildenafil	--	--	--	No concerns	Major concerns	No concerns	No concerns	High	

Comparison	Number of Studies	Within-study bias	Reporting bias	Indirectness	Imprecision	Heterogeneity	Incoherence	Confidence rating	Reason(s) f downgradi
**Beta blockers vs Vericiguat 10mg**	--	--	--	No concerns	No concerns	Major concerns	No concerns	Moderate ▼	|
**Beta blockers vs Vericiguat 15mg**	--	--	--	No concerns	Major concerns	No concerns	No concerns	Moderate ▼	|
**Digoxin vs MRA**	--	--	--	No concerns	Major concerns	No concerns	No concerns	Moderate ▼	|
**Digoxin vs Sildenafil**	--	--	--	No concerns	Major concerns	No concerns	No concerns	Moderate ▼	|
**Digoxin vs Vericiguat 10mg**	--	--	--	No concerns	Major concerns	No concerns	No concerns	Moderate ▼	|
**Digoxin vs Vericiguat 15mg**	--	--	--	No concerns	Major concerns	No concerns	No concerns	Moderate ▼	|
**MRA vs Sildenafil**	--	--	--	No concerns	Major concerns	No concerns	No concerns	Moderate ▼	|
**MRA vs Vericiguat 10mg**	--	--	--	No concerns	Major concerns	No concerns	No concerns	Moderate ▼	|
**MRA vs Vericiguat 15mg**	--	--	--	No concerns	Major concerns	No concerns	No concerns	Moderate ▼	|
**Sildenafil vs Vericiguat 10mg**	--	--	--	No concerns	Major concerns	No concerns	No concerns	Moderate ▼	|
**Sildenafil vs Vericiguat 15mg**	--	--	--	No concerns	Major concerns	No concerns	No concerns	Moderate ▼	|
