# Supplementary material for: Comparison of Pharmacological Treatment Effects on Long-Time Outcomes in Heart Failure With Preserved Ejection Fraction: A Network Meta-analysis of Randomized Controlled Trials
Source: Front Pharmacol. 2021 Nov 24;12:707777. doi: 10.3389/fphar.2021.707777 (PMC8652335; doi:10.3389/fphar.2021.707777)
Supplement: Supplementary file 5 [file DataSheet2.doc]

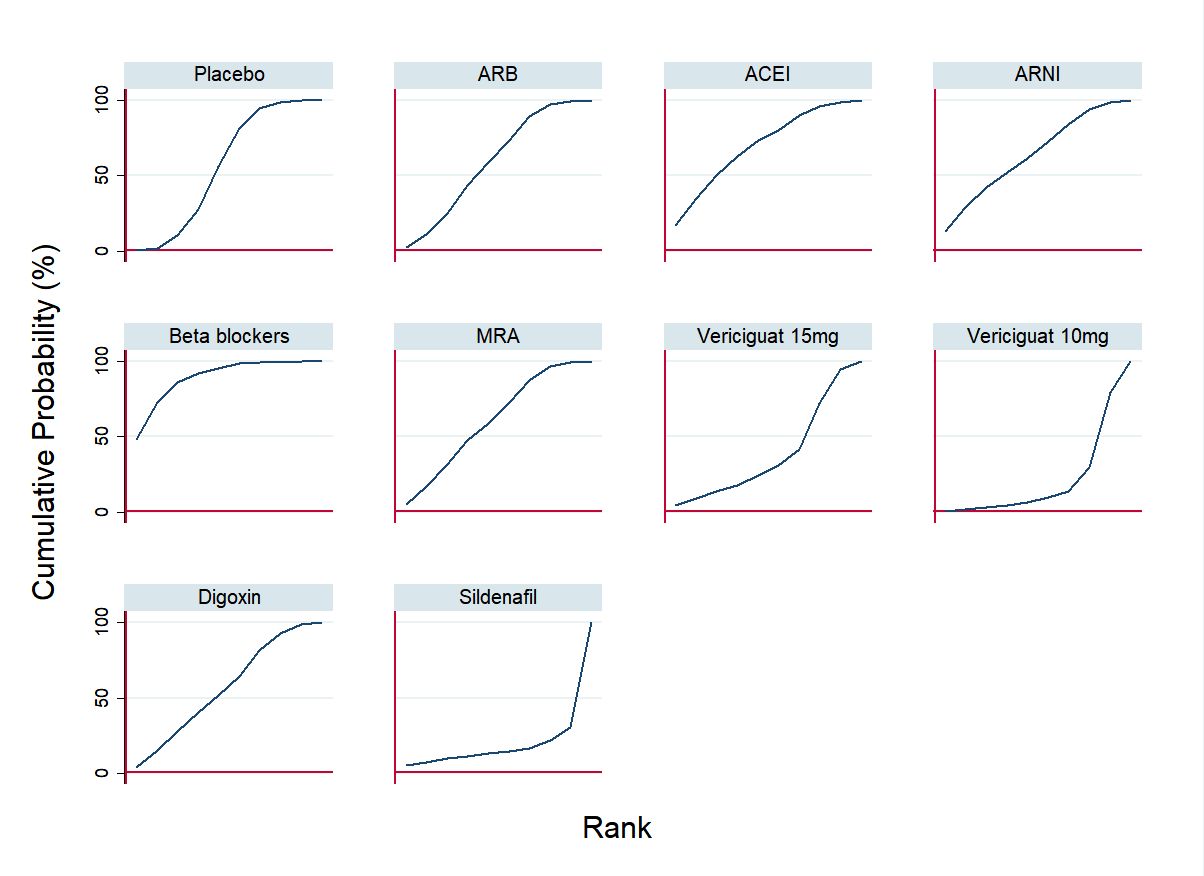


Supplementary Figure 1. Treatment strategy for all-cause mortality: SUCRA rankogram plots

ACEI, angiotensin-converting enzyme inhibitor; ARB, angiotensin receptor blocker; ARNI, angiotensin receptor neprilysin inhibitor; MRA, mineralocorticoid receptor antagonist


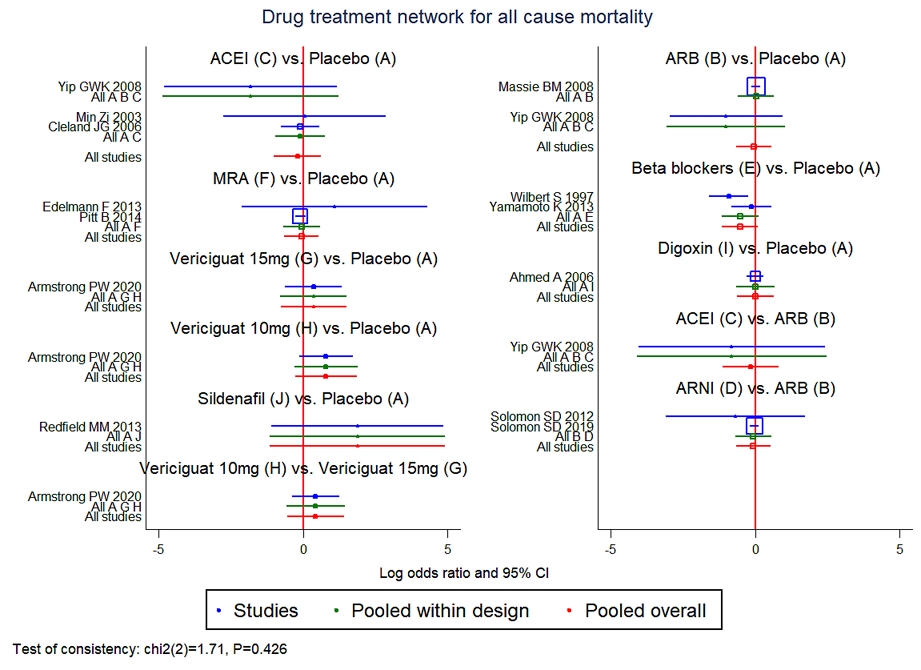


Supplementary Figure 2. All-cause mortality (primary outcome): Network plot - All trials


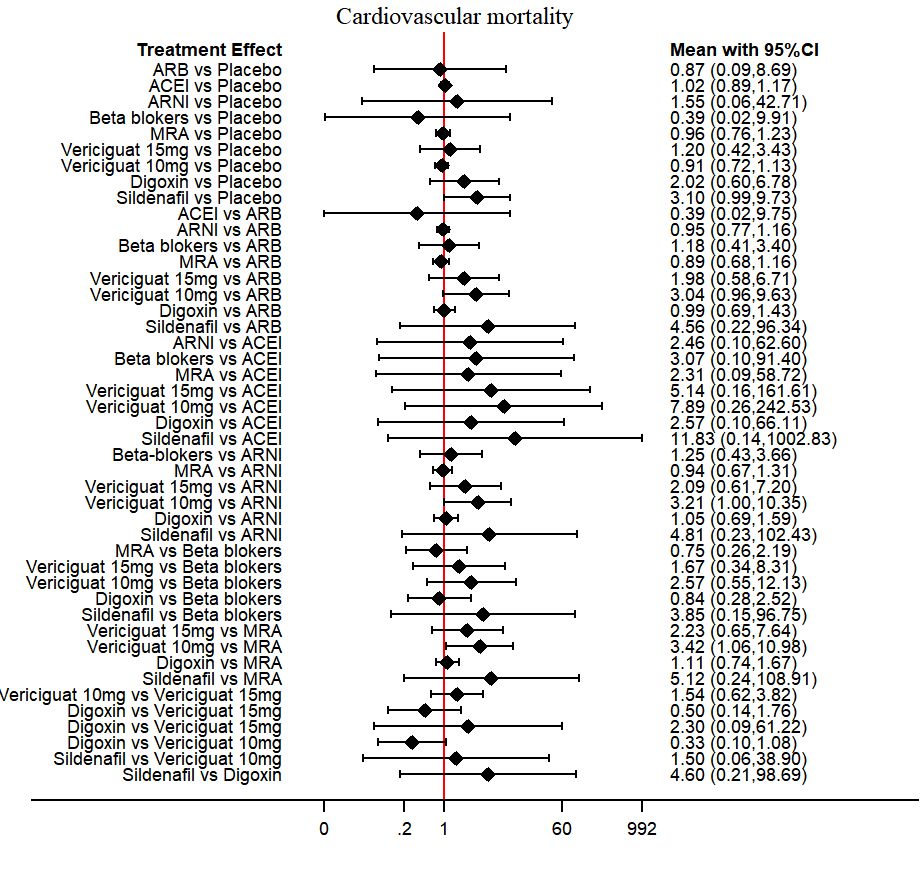


Supplementary Figure 3. Cardiovascular mortality (secondary outcome): Forest plot (estimates as hazard ratio) - All trials


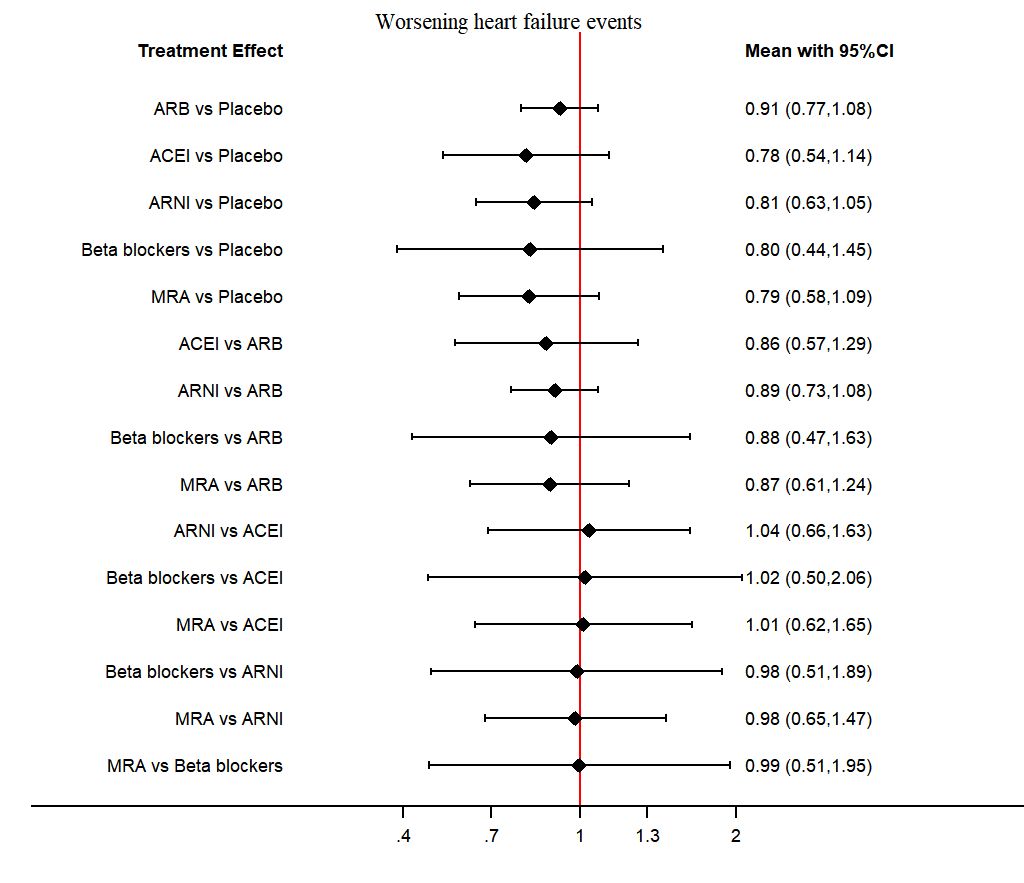


Supplementary Figure 4. Worsening heart failure events (secondary outcome): Forest plot (estimates as hazard ratio) - All trials


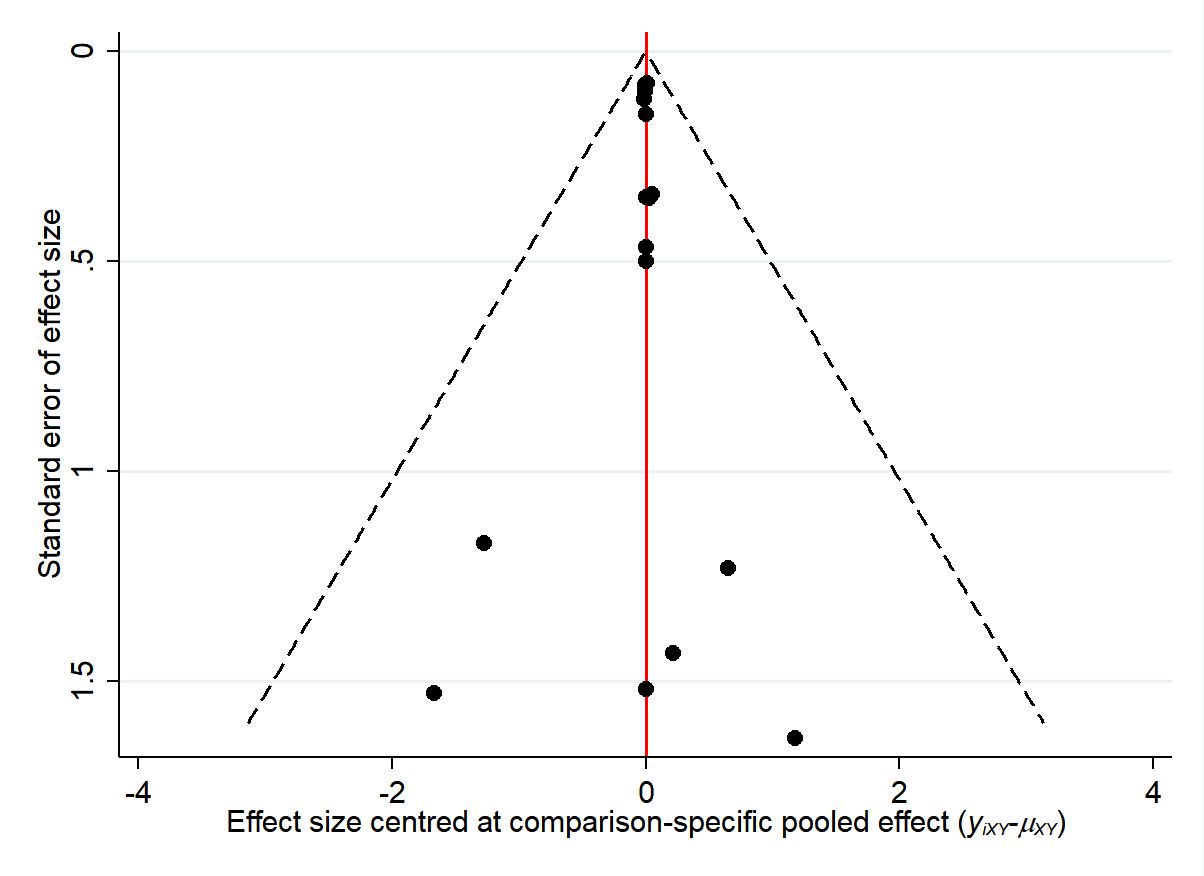


Supplementary Figure 5. Assess of risk of bias of the included studies


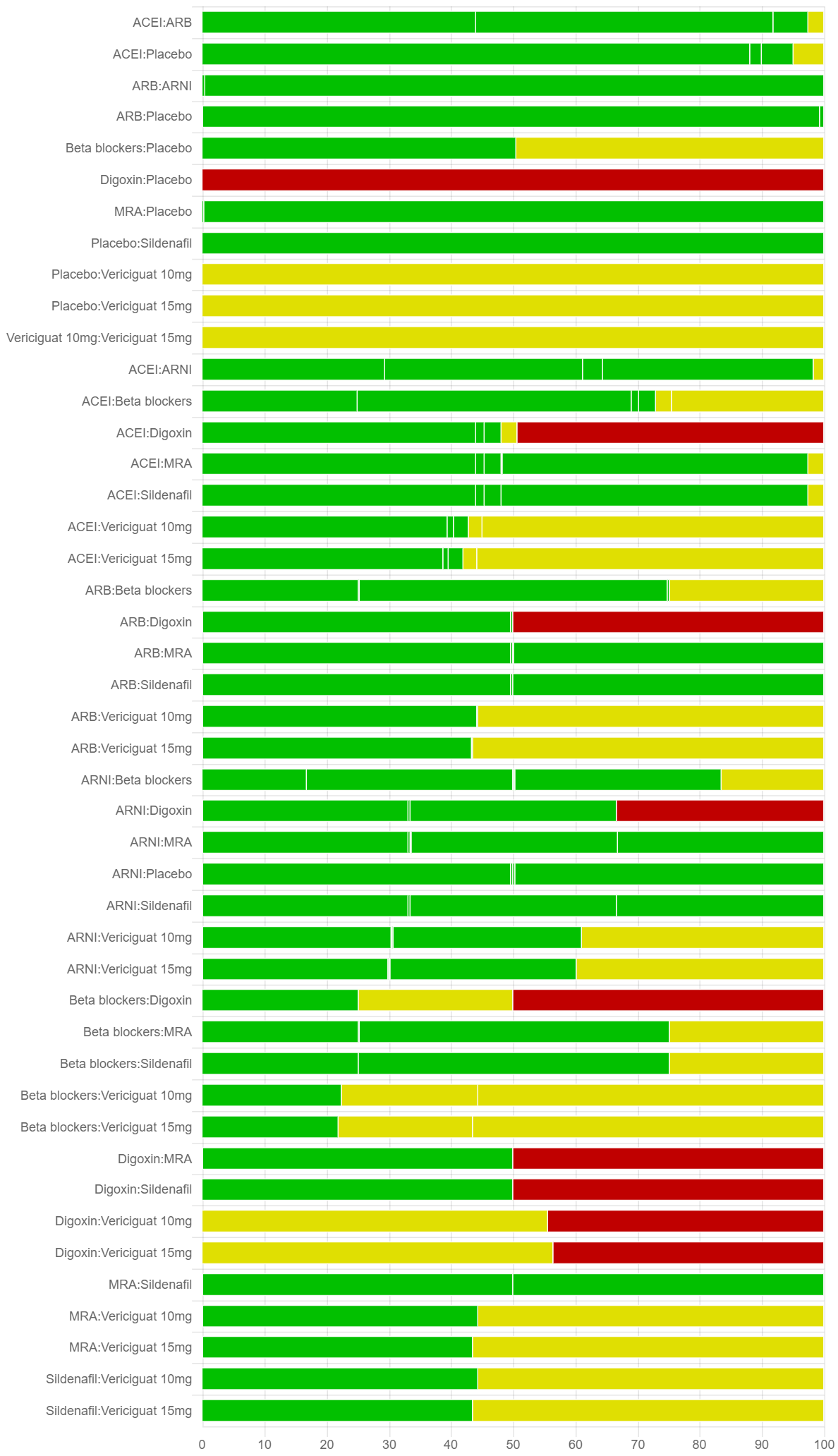


Supplementary Figure 6. The bar chart shows the contributions of each piece of study to the network estimate
